# Single-cell transcriptomics reveal distinct immune-infiltrating phenotypes and macrophage–tumor interaction axes among different lineages of pituitary neuroendocrine tumors

**DOI:** 10.1186/s13073-024-01325-4

**Published:** 2024-04-24

**Authors:** Shaojian Lin, Yuting Dai, Changxi Han, Tianyi Han, Linfeng Zhao, Renyan Wu, Jianyue Liu, Bo Zhang, Ning Huang, Yanting Liu, Shujing Lai, Jintong Shi, Yu Wang, Meiqing Lou, Jing Xie, Yijun Cheng, Hao Tang, Hong Yao, Hai Fang, Yan Zhang, Xuefeng Wu, Lei Shen, Youqiong Ye, Li Xue, Zhe Bao Wu

**Affiliations:** 1 Department of Neurosurgery, Center of Pituitary Tumor, Ruijin Hospital, Shanghai Jiao Tong University School of Medicine, Shanghai, China; 2 Department of Neurosurgery, Center for Immune-Related Diseases at Shanghai Institute of Immunology, Ruijin Hospital, Shanghai Jiao Tong University School of Medicine, Shanghai, China; 3 Department of Neurosurgery, First Affiliated Hospital of Wenzhou Medical University, Wenzhou, China; 4 Shanghai Institute of Hematology, State Key Laboratory of Medical Genomics, National Research Center for Translational Medicine at Shanghai, Rujin Hospital, Shanghai Jiao Tong University School of Medicine, Shanghai, China; 5 Shanghai Key Laboratory of Tumor Microenvironment and Inflammation, Shanghai Jiao Tong University School of Medicine, Shanghai, China; 6 Shanghai Institute of Immunology, Department of Immunology and Microbiology, Shanghai Jiao Tong University School of Medicine, Shanghai, China; 7 Department of Neurosurgery, Ren Ji Hospital, Shanghai Jiao Tong University School of Medicine, Shanghai, China; 8 Department of Neurosurgery, Shanghai General Hospital, Shanghai Jiao Tong University School of Medicine, Shanghai, China; 9 Department of Pathology, Ruijin Hospital, Shanghai Jiao Tong University School of Medicine, Shanghai, China; 10 Med-X Research Institute and School of Biomedical Engineering, Shanghai Jiao Tong University, Shanghai, China; 11 Shanghai Institute of Immunology, Department of Immunology and Microbiology and the Ministry of Education Key Laboratory of Cell Death and Differentiation, Shanghai Jiao Tong University School of Medicine, Shanghai, China; 12 Shanghai Institute of Immunology, Department of Immunology and Microbiology, State Key Laboratory of Systems Medicine for Cancer, Shanghai Jiao Tong University School of Medicine, Shanghai, China; 13 Shanghai Center for Brain Science and Brain-Inspired Technology, Shanghai, China

**Keywords:** Pituitary neuroendocrine tumors, Single-cell RNA sequencing, Immune microenvironment subtypes, Tumor-associated macrophage, *CX3CR1*^+^ macrophage, INHBA-ACVR1B axis

## Abstract

**Background:**

Pituitary neuroendocrine tumors (PitNETs) are common gland neoplasms demonstrating distinctive transcription factors. Although the role of immune cells in PitNETs has been widely recognized, the precise immunological environment and its control over tumor cells are poorly understood.

**Methods:**

The heterogeneity, spatial distribution, and clinical significance of macrophages in PitNETs were analyzed using single-cell RNA sequencing (scRNA-seq), bulk RNA-seq, spatial transcriptomics, immunohistochemistry, and multiplexed quantitative immunofluorescence (QIF). Cell viability, cell apoptosis assays, and in vivo subcutaneous xenograft experiments have confirmed that INHBA-ACVR1B influences the process of tumor cell apoptosis.

**Results:**

The present study evaluated scRNA-seq data from 23 PitNET samples categorized into 3 primary lineages. The objective was to explore the diversity of tumors and the composition of immune cells across these lineages. Analyzed data from scRNA-seq and 365 bulk RNA sequencing samples conducted in-house revealed the presence of three unique subtypes of tumor immune microenvironment (TIME) in PitNETs. These subtypes were characterized by varying levels of immune infiltration, ranging from low to intermediate to high. In addition, the NR5A1 lineage is primarily associated with the subtype characterized by limited infiltration of immune cells. Tumor-associated macrophages (TAMs) expressing *CX3CR1*^+^*, C1Q*^+^*,* and *GPNMB*^+^ showed enhanced contact with tumor cells expressing *NR5A1* + *, TBX19*^+^*,* and *POU1F1*^+^, respectively. This emphasizes the distinct interaction axes between TAMs and tumor cells based on their lineage. Moreover, the connection between *CX3CR1*^+^ macrophages and tumor cells via INHBA-ACVR1B regulates tumor cell apoptosis.

**Conclusions:**

In summary, the different subtypes of TIME and the interaction between TAM and tumor cells offer valuable insights into the control of TIME that affects the development of PitNET. These findings can be utilized as prospective targets for therapeutic interventions.

**Supplementary Information:**

The online version contains supplementary material available at 10.1186/s13073-024-01325-4.

## Background

Pituitary neuroendocrine tumors (PitNETs) are a frequently occurring form of intracranial brain tumor. The estimated prevalence rate of clinically significant PitNETs is approximately 1 case per 1000 individuals [1]. About 2% of cases display aggressive behavior, while up to 15% show invasion, with a few cases of craniospinal or distant metastases occurring in 0.1–0.2% of cases. PitNETs can be either functional (i.e., producing hormones and causing hormonal imbalances) or non-functional, leading to neurological symptoms based on size and location. Although significant therapeutic progress in strategies, such as medication and surgery, has been made, many PitNET patients have poor clinical outcomes. The 2022 World Health Organization classification reported the role of key transcription factors in the differentiation of distinct types of pituitary cells, indicating the complexity and heterogeneity of PitNETs [2]. Revolutionary advances in single-cell RNA sequencing (scRNA-seq) and bulk-RNA sequencing technology allow a more comprehensive understanding of the origin and differentiation status of PitNET cells [3–9]. In addition to deciphering the tumor cells, efforts have been made to characterize the tumor immune microenvironment (TIME), improving the clinical benefit of immunotherapy, especially anti-PD-1/L1 and anti-CTLA4 antibodies [10, 11]; however, many patients still have poor outcomes [12, 13]. Therefore, a deeper insight into PitNET TIME and identifying potential targets is crucial.

TIME is a well-recognized factor in the development and progression of PitNETs [14–16]. Macrophages and T lymphocytes are the most prevalent infiltrating immune cells in PitNETs [17, 18]. Also, other immune cell subtypes such as B lymphocytes, *FOXP3*^+^ cells, neutrophils, NK cells, or mast cells may be present less frequently [15]. However, the specific function and heterogeneity of these immune cell subtypes in PitNETs remain poorly understood, which can help identify new therapeutic targets. Some studies have endeavored to characterize the immune microenvironment of PitNETs using immunohistochemistry (IHC) [16], flow cytometry (FC) [19], and RNA-seq (including both bulk RNA-seq and single-cell RNA-seq) [3, 5–7, 9]. Macrophages, a highly heterogeneous group of immune cells, have many functions, including phagocytosis, antigen presentation, cytokine production, and tissue remodeling [20]. Growing evidence has suggested that immune cells, particularly macrophages, play an essential role in the development and progression of pituitary adenomas [21, 22]. Macrophages are present in both normal pituitary and PitNETs, and their types and populations vary depending on the subtype of PitNETs [23–25]. M2 macrophages are prevalent in the pituitary gland and may play a role in tumorigenesis [26]. The number of macrophages correlates with the size and invasiveness of PitNETs; higher macrophage content is seen in sparsely granulated somatotrophinomas and null-cell PitNETs compared to densely granulated somatotrophinomas or corticotrophinomas [22]. M2 macrophages are also more prevalent in non-functioning PitNETs (NF-PitNETs) with cavernous sinus invasion than in non-invasive NF-PitNETs. A higher M2/M1 macrophage ratio was associated with larger and more proliferative NF-PitNETs [25–27]. Moreover, the M2 macrophage-conditioned medium increased the proliferation, invasion, and migration of primary NF-PitNET cells [19, 27, 28]. However, the heterogeneity of macrophages in various PitNETs and their subpopulation classification and communication with tumor cells remain unexplored.

Here, we performed integrated analysis on scRNA-seq and bulk-RNA seq data of 3 tumor lineages from 23 adult humans to establish the transcriptional landscape of immune cells in PitNETs. The pituitary tumors were classified into three immune infiltration states based on immune infiltration, and five subtypes of related macrophages were identified. IHC by TMA (tissue microarray) staining and FC confirmed that POU1F1 lineages had more immune cells, while NR5A1 lineages had lower immune cell infiltration. *CX3CR1*^+^, *LYVE1*^+^, *C1Q*^+^*,* and *GPNMB*^+^ macrophages in PitNETs were identified by multiplex IHC (mIHC). Furthermore, INHBA secreted by *CX3CR1*^+^ macrophages partially induced cell apoptosis. In contrast, blocking the INHBA receptor ACRV1B reversed this effect. Overall, our findings provide a comprehensive atlas of PitNETs TIME subtypes, particularly from the aspect of macrophages, and highlight the role of *CX3CR1*^+^ macrophages in modulating transcriptional status and intercellular communication patterns.

## Methods

### Clinical samples

Patient recruitments three cohorts of patients were recruited: 23 scRNA-seq, 365 bulk RNA-seq PitNETs, 128 Immunohistochemistry (IHC) of TMA, and 45 flow cytometry for analysis and validation. All patients received surgery at the Department of Neurosurgery at Ruijin Hospital, an affiliate of Shanghai Jiao Tong University School of Medicine. The scRNA-seq and flow cytometry PitNETs cohort underwent surgery between 2020 and 2023, while the bulk RNA-seq and IHC PitNETs cohorts were collected between 2017 and 2022. Both cohorts only included subjects without previous malignancies. The clinical data of all patients were retrospectively obtained from medical records (Additional file 2: Table S1-5). The patients are assigned numbers matching the GroupsIndex column in Supplementary Table S1-5. Written informed consent was obtained from all patients, and the Ruijin Hospital ethical committee approved the study.

### Tissue dissociation and primary tumor cell extraction

Tissues were transported in DMEM (Gibco, Cat. no. 11875–093) with 1 mM protease inhibitor (Solarbio, Cat. no. P6730) on ice to preserve viability, washed two to three times with phosphate-buffered saline (PBS; Hyclone, Cat. no. SH30256.01), then minced on ice. We used dissociation enzyme cocktail 1 mg/ml Type VIII Collagenase (Sigma-Aldrich, Cat. no. C2139), 2 mg/ml Dispase II (Sigma-Aldrich, Cat. no.4942078001), 1 mg/ml Trypsin Inhibitor (Sigma-Aldrich, Cat. no. T6522), and 1 unit/ml DNase I (NEB, Cat. no. M0303S) dissolved in serum-free DMEM to digest the tissues. Neoplastic tissues were dissociated at 37 °C with a shaking speed of 50 r.p.m for about 40 min. We repeatedly collected the dissociated cells at intervals of 20 min to increase cells yield and viability. Cell suspensions were filtered using a 40-μm nylon cell strainer (Falcon, Cat. no. 352340) and red blood cells (RBC) were removed by RBC lysis buffer (Invitrogen, Cat. no. 1966634) with 1 unit/ml DNase I. Dissociated cells were washed with PBS containing 0.04% bovine serum albumin (BSA; Sigma-Aldrich, Cat. no. B2064) with step-by-step descending centrifuging speed and increasing time. Cells were stained with 0.4% Trypan blue (Invitrogen, Cat. no. T10282) to check the viability and then cultured in DMEM containing 10% FBS and 1% antibiotic mixture.

### RNA sequencing alignment

To quantify gene expression in the transcriptome, the RNA sequencing raw FASTQ files were aligned to the human reference genome GRCh38 (release 40). The human reference genome and its annotation file were acquired from the GENCODE database, accessible at https://www.gencodegenes.org/. Salmon (v1.8.1) [29] was used to generate the count and transcripts per kilobase of exon model per million mapped reads (TPM) matrix. The transcript counts were combined using DESeq2 [30] and then transformed into fragments per kilobase million (FPKM) to normalize the gene length, using the TPM matrix to evaluate the gene expression level. To identify differentially expressed genes, we used the limma [31] package with a significance level set at an adjusted *P*-value < 0.05 and a log2-transformed fold change (log2FC) > 0.58 (FC > 1.5). To identify differentially expressed genes between more than two conditions, we used analysis of variance (ANOVA) to calculate the *P*-values.

### Functional enrichment analysis

Gene Set Enrichment Analysis (GSEA) [32] was employed with the pre-ranked algorithm and the R package clusterProfiler [33] to perform functional enrichment analysis. Genes were ranked based on their log2FC. The gene sets from the Molecular Signatures Database (MSigDB, v7.5.1) [34] of the Broad Institute were used. Specifically, we used the HALLMARK gene sets (H) [34], which represent 50 well-defined biological processes, and the Kyoto Encyclopedia of Genes and Genomes (KEGG) [35, 36] database to identify differences induced by various drugs. A significance cutoff of *P*-value < 0.05 was considered. To visualize the enrichment results, we utilized the enrichplot R package (https://bioconductor.org/packages/enrichplot/). Another functional enrichment analysis algorithm, Enrichr (https://maayanlab.cloud/Enrichr/) [37] web service, was conducted to evaluate gene signatures. *P*-value < 0.05 was set to the significance level.

### Single-cell RNA sequencing alignment and generation of gene expression matrix

Raw sequencing reads of the cDNA library was processed through the BD Rhapsody Whole Transcriptome Assay Analysis Pipeline (v1.8), which included filtering by reads quality, annotating reads, annotating molecules, determining putative cells, and generating a single-cell expression matrix. The pipeline also selected the sample origin of every single cell via the sample determination algorithm according to the sequencing reads of the SampleTag library. Among all the output files, a matrix of UMI counts for each gene per cell was used for downstream analysis. Genome Reference Consortium Human Build 38 (GRCh38) was used as a reference for the BD pipeline.

### Analysis of single-cell expression data and cell type annotation

Gene expression matrices were imported for downstream analysis using the R Seurat package [38].

#### Single-cell quality control (Additional file 2: Table S6)

In the initial step, we conducted a filtration process to eliminate cells exhibiting low gene expression. The gene expression matrices of individual cells were imported for subsequent analysis using the R Seurat package. Cells with expressed genes < 200, or unique counts > 50,000 or < 500, or expressed mitochondrial RNA > 30% were removed for quality control, resulting in 98,523 cells being retained. Upon analyzing the cell distributions across the 23 samples, the median number of captured cells was 2837. Among the 23 samples, 6 had a cell count exceeding 5000, with 2 samples notably containing a high number of captured cells (> 10,000 cells). To ensure effective integration, we employed random downsampling to limit the maximum number of enrolled cells for each sample to 5000 using the R sample function. Ultimately, a total of 69,539 cells from PitNETs were included for downstream data analysis (Additional File 2: Table S2). We performed the doublets analysis by using the R package DropletUtils (https://bioconductor.org/packages/DropletUtils/); we identified 108 cells (0.15%) as potential doublets among the total 69,539 cells. 

#### Batch effect adjustment

Following the generation of the Seurat object containing 69,539 cells, we identified the top 3000 highly variable genes and utilized them to conduct principal component analysis (PCA). Batch effect correction across samples was executed using the R package Harmony [39], with the parameter “max.iter.harmony” set to 5. Subsequently, the top 20 harmony coordinates were chosen for clustering analysis and dimensionality reduction.

#### Clustering and dimensionality reduction

The top 20 harmony coordinates were selected for graph-based unsupervised cell clusters with the resolution was set to 0.8. Dimensionality reduction methods t-Distributed Stochastic Neighbor Embedding (t-SNE) [40] and Uniform Manifold Approximation and Projection (UMAP) [41] was performed for visualization. The t-SNE was selected for final interpretation.

#### Annotation of major cell populations

After clustering, 30 clusters were calculated. Marker genes for each cluster were calculated using the “FindAllMarkers” function under the following criteria: log_2_ fold changes (log_2_FC) > 0.25, min.pct > 0.1, and adjusted *P* < 0.05. Cells were annotated using both machine-learning-based software SingleR [42] and high expression of canonical markers (i.e., *EPCAM* for epithelial cells, *NCAM1* for neuron cells, *VWF* for endothelial cells, *DCN* for fibroblast. *PTPRC* for immune cells, *C1Q* for macrophage/Dendritic-cells (MDC), and *CD3* for T cells, *CD79A* for B cells) in each cluster. For the second step of cell annotation, the immune populations were extracted separately and performed the entire workflow of normalization, batch effect correction, unsupervised cell clustering, and cell annotation and identification. The small cell population inner the immune cell populations were finally identified.

### Identification of tumor cells in PitNETs

The tumor cells’ identification was described as (i) expression of neuron markers (*NCAM1*) and canonical markers of PitNETs, such as *POU1F1*, *TBX19,* and *NR5A1* and (ii) higher copy number variation level. The copy number karyotyping analysis was performed by inferCNV (https://github.com/broadinstitute/infercnv) and CopyKAT (https://github.com/navinlabcode/copykat) [43] package with default parameter.

### Differentially expressed genes calculation and transcription factors activities estimation

We employed Seurat's “FindMarkers” feature to detect genes expressed at varying levels between separate cell types. The log fold change (logFC) threshold was set at 0.01, and the minimum percentage of cells expressing the gene (min.pct) was set at 0.01. The significance of DEGs was determined by log2FC > 0.5 and adjusted *P*-value < 0.05. Protein activities of transcription factors (TFs) were estimated using DoRothEA [44]. A significance criterion of *P* < 0.05 was employed to identify variations in TF activity across various cell types.

### Generation of cell signatures based on scRNA-seq

The top 200 expressed genes (log_2_FC > 0.58 and adjusted *P*-value < 0.05) in each cell type were selected as an in-house cell marker database of PitNETs, which was enrolled for analysis on bulk RNA-seq data. The enrichment score to infer cell abundance from bulk RNA-seq was calculated using a single sample gene set enrichment analysis (ssGSEA) algorithm via the R GSVA [45] package.

### Trajectory analysis of macrophages

In order to assess the possible dynamic changes in cell state across several subclusters of tumor-associated macrophages (TAM), we employed trajectory analysis using the R package Monocle2 (version 2.24.0) [46]. The DDRTree method was used to reduce dimensionality based on the top 50 DEGs in each TAM subcluster and monocyte. Based on the trajectory results, monocytes were defined as the initiating point of the trajectory. Genes with branch-dependent (*CX3CR1*^+^ macro and *C1Q*^+^ macro) expressions were identified using the BEAM subprogram, filtered with a *q*-value < 0.05, and visualized by plot_genes_branched_heatmap function. ScTour and DifussionMap were utilized with the default parameters.

### Cell–cell communication analysis

We applied the R package Cellchat to evaluate the interaction weights between tumor cells and TAMs [47]. First, we created a cell chat object with default parameters. The ligand–receptor interaction database we used for analysis was “CellChatDB.human” without additional supplement.

NicheNet was applied to infer the interaction mechanisms between TAMs and malignant cells [48]. We defined the niches of interest for every subcluster. Clustered cells with gene expression over 10% were considered for ligand and receptor interactions. The top 100 ligands and top 1000 targets of differentially expressed genes of “senders” and “receivers” were extracted for paired ligand–receptor activity analysis. When evaluating the regulatory network of TAMs on tumor cells, NR5A1 + tumor cells were considered “receivers”.

In contrast, *POU1F1*^+^ tumor cells and *TBX19*^+^ tumor cells were used as reference cells to check the regulatory potential of TAMs on tumor cells. The ligand_activity_target_heatmap in Nichenet_output was used to show the regulatory activity of ligands. Activity scores ranged from 0 to 1.

### Spatial transcriptomics analysis

The stereo-seq data is preprocessed using the STOmics Analysis Workflow (SAW). The read mapping pipeline uses STAR to align the sequenced reads to the human reference genome (GRCh38). Reads for which mass value (*Q* = − 10log(err rate)) is less than 15 account for more than 40% of the total number of bases, or *N* reads with more than 5 bases or containing linker sequences were filtered. Valid CID sequence must completely match chip barcode sequence. The gene quantification pipeline uses Bam2Gem to count the number of reads mapped to each gene. Bin200 was set as the basic unit for further data statistics.

The SpatialFeaturePlot function in Seurat generated spatial feature expression plots (Additional file 2: Table S7). Signature scores were integrated into the “metadata” of the spatial transcriptomics (ST) dataset by calculating the mean expression levels of each gene from the scRNA-seq dataset. The estimation of cellular composition for the spatial transcriptomics spots was performed using the SpatialDecon function, and pie plots visualizing the deconvolution results were generated using the DeconPieplot function in the Cottrazm package (version 0.1.1) [49].

### Immunohistochemistry staining of tissue microarray

The tissue microarray (TMA) dataset (*n* = 128, Additional file 2: Table S3) was used to evaluate the immune cell infiltration. Following deparaffinization and rehydration, heat-induced epitope retrieval (HIER) was performed by submerging the slides in antigen unmasking solution (Solarbio).

After blocking endogenous peroxidase and nonspecific binding sites (0.3% H2O2 and 5% normal goat serum, sequentially), primary antibodies were applied at 4 °C overnight. Slides were incubated with Dako REAL EnVision HRP rabbit/mouse (belong to K5007, DAKO, Glostrup, Denmark) at RT for 20 min, followed by treatment with Dako REAL DAB + CHROMOGEN and Dako REAL substrate buffer (belong to K5007, DAKO, Glostrup, Denmark) to visualize staining signals under light microscopy, finally counterstained using hematoxylin solution. Stained slides were scanned using Ocus (Grundium, Tampere, Finland) and analyzed with Qupath software (see below).

### IHC image analysis

Stained slides were scanned using Ocus (Grundium, Tampere, Finland) and analyzed with Qupath software v0.3.0. The built-in stain vector estimator preprocessed images. Cells with shape and stain parameters in each area were identified by build-in cell detection via nucleus stain (hematoxylin). The threshold for positive mean DAB optical density (OD) was determined based on the staining pattern and intensities observed in all photos for each antibody. The percentage of CD45, CD68, CD8, Ki67, and Cleaved Caspase 3 positive cells were quantified using a customized cellular multiplex algorithm. Scripts were produced for the analysis methodology of all the above slide pictures. These scripts were then executed in batches for each set of images and subsequently reviewed by two pathologists who are experts in the field. All quantifications were evaluated blinded to patient clinical information and outcomes.

### Multiplex immunohistochemistry and analysis

Formalin-fixed and paraffin-embedded tissue Sects. (3 mm) were de-paraffinized and rehydrated. Next, heat-induced epitope retrieval (HIER) was performed, followed by blocking with 3% hydrogen peroxide in TBST for 10 min and staining with the multiplex mIHC kit (PerkinElmer, NEL861001KT, Shanghai Kelin Institute). Briefly, after the first primary antibody staining, slides were incubated using the HRP-polymer detection system for 10 min, then visualization using Opal TSA working solution (1:100) for another 10 min. Afterward, antigen retrieval was conducted again to prepare the slides for the next antibody. Using this Opal staining method, primary antibodies were applied sequentially. Lastly, slides were counterstained with DAPI (Sigma, 1:1000) for nuclei visualization and subsequently coverslipped using the Hardset mounting media (VectaShield, H-1400).

All tissue sections that underwent multiplex fluorescent staining for each fluorophore were imaged using the Vectra Polaris imaging system (PerkinElmer, Shanghai Kelin Institute) under the appropriate fluorescent filters to produce the spectral library required for multispectral analysis. A whole slide scan of the multiplex tissue sections produced multispectral fluorescent images visualized in Phenochart (PerkinElmer).

### Cell culture and reagents

The GH3, AtT20, MMQ, and RC-4BC cell lines were purchased from the American Type Culture Collection (ATCC, Manassas, VA, USA). GH3, AtT20, and MMQ cell line Twere cultured in Ham's F-12 K medium (L450KJ, BasalMedia) supplemented with 2.5% FBS (S615JY, BasalMedia), 15% horse serum (26,050,088, ThermoFisher), and 1% penicillin/streptomycin (C100C5, NCM biotech. RC-4BC cells were cultured in DMEM (L130KJ, BasalMedia) with 10% FBS, 5 ng/ml recombinant rat EGF (ab290070, Abcam), and 1% penicillin/streptomycin. The following inhibitor and recombinant protein were used: recombinant INHBA (C687, Novoprotein), SB-505124 (HY-13521, MedChemExpress), A 83–01 (HY-10432, MedChemExpress), and recombinant human Follistatin (10685-H08H, Sino Biological).

### Flow cytometry and cell sorting

For surface staining, cells were resuspended in 50 μl of PBS containing antibody cocktails and stained at room temperature in the dark for 30 min. Antibodies used for flow cytometry are listed (Additional file 2: Table S8). For intracellular staining, cells were fixed and permeabilized by Foxp3 Fixation/Permeabilization kit (Thermo Fisher Scientific) at 4 °C for 45 min and stained with 50 μl of 1 × permeabilization buffer containing antibody cocktails at 4 °C in the dark for 45 min.

For cell sorting, among the immune cells from human pituitary tumor cell suspensions, CD45-positive were divided into T cells (CD45 + CD3 +), B cells (CD45 + CD3-CD19 +), NK cells (CD45 + CD3-CD19-CD56 +), and macrophages (CD45 + CD3-CD19-CD56-CD11b +); among the CD45-negative, non-immune cells were divided into epithelial cells (CD45-EPCAM + CD31-), endothelial cells (CD45-CD31 + EPCAM-), and stromal cells (CD45-CD31-EPCAM-CD90 +). Among CD14-macrophages, CX3CR1 + , C1Q + , and Lyve + were used to classify macrophages, respectively. The gating strategy is shown in Additional file 1: Fig S11.

All flow data were acquired by BD FACSDiva software v8.0.2 and analyzed by FlowJo VX.

### TAM and tumor cell co-culture system

We isolated *CX3CR1*^+^ TAMs from three independent patients (334, 345, and 356) belonging to the SF1 lineage. The clinical details for these patients are provided in Supplementary table s3. We co-cultured the sorted *CX3CR1*^+^ TAMs with matched CD45- tumor cells. Briefly, 1 × 10^4^ TAMs were sorted and seeded onto Transwell polycarbonate filters (0.4 µm pore, 6.5 mm membrane diameter; Corning Incorporated, Corning, NY, USA). Simultaneously, 5 × 10^5^ primary tumor cells were seeded into the lower compartments of Transwell chambers, allowing co-culture with TAMs in the upper compartment for 72 h. The initially identified tumor cells from the lower compartment were then collected for further investigation.

### CellTiter-Glo luminescence assay

CellTiter-Glo luminescence assay (Promega, Madison, WI) was used to determine the suppressive effect of the corresponding drug on the control or gene-edited cell line. The cells were distributed at 2000 cells per well on a 96-well plate. The cells were exposed to the appropriate drugs in a 10% FBS-F12K medium at the prescribed doses. Cells were cultured for the indicated time before adding 100 μL of the CellTiter-Glo® luminescence assay reagent in each well. Cells were incubated for an additional 10 min at room temperature to stabilize luminescent signals and transferred to 96-well black plates. Measurements were performed using a luminescence reader (TECAN, Männedorf, Switzerland). Data was analyzed by GraphPad Prism 5 and normalized to the control group. The* p*-value was calculated using the Student's *t*-test. Data was generated from at least three independent experiments.

### Cell apoptosis

Flow cytometric analysis was conducted using the Annexin V, FITC Apoptosis Detection Kit (AD10, Dojindo) to evaluate cellular apoptosis. Primary tumor cells were harvested and rinsed twice with PBS. Subsequently, the cells were incubated with Annexin V-FITC/PI per the manufacturer’s instructions. The cells were acquired and analyzed using BD FACS Calibur with FlowJo (version 7.6.1) software.

### Immunofluorescence and analysis

The cells were derived from samples 375, 381, and 382. Sorted 1 × 10^4^ *CX3CR1*^+^ and *CX3CR1*^*−*^ macros were centrifuged on a slide by Thermo Scientific Shandon Cytospin. After that, cells were washed once with PBS and then fixed with 4% paraformaldehyde for 5 min at room temperature. Cells were washed thrice with PBS and permeabilized with 0.3% Triton-X for 5 min at room temperature. After three times PBS washing, cells were blocked at room temperature with 5% FBS blocking solution for 30 min. The cells were incubated overnight at 4℃ with anti-INHBA and anti-CD68 primary antibodies in a blocking solution. The cells were then washed for 5 min at room temperature three times with PBS. The cells were then incubated with secondary antibodies for 1 h at room temperature.

Anti-goat FITC for INHBA and anti-rabbit Corelite 594 for CD68 were used. All secondary antibodies were diluted in PBS. After that, the cells were washed at room temperature three times with PBS and stained with Hoechst at 1/1000 (40731ES10, yeasen) for 5 min at room temperature. Finally, the slides were washed three times with PBS and mounted on glass slides with AntiFade Mounting Medium ( G1401-5ML, Servicebio). Image acquisition was performed with an Axio Imager M2 (Carl Zeiss Ltd) and an Apotome. The Zen software piloted 2 (363 oil immersion objective) (Carl Zeiss Ltd). Additional file 2: Table S8 summarizes the antibodies used and how they were diluted.

To quantify the fluorescence intensity, we used ImageJ software with default parameters. The grayscale value assigned to each pixel in the single-channel fluorescence image reflects the corresponding fluorescence intensity. The *INHBA* mean fluorescence intensity (MFI) was determined using the formula: Mean Fluorescence Intensity (MFI) = Total Fluorescence Intensity in the Region / Area of the Region. The grayscale value and the corresponding area were determined for each region, and the MFI for each photo was calculated. Statistical analysis was performed using a Student’s *t*-test.

### Real-time RT-PCR

Total RNA was extracted from tissue samples and cells using TRIzol reagent (AG21102, Accurate Biology) after washing with PBS. According to the manufacturer’s instructions, cDNA was synthesized from purified RNA using an Evo M-MLV RT Mix Kit with gDNA Clean for qPCR (AG11728, Accurate Biology). SYBR Green PCR Master Mix (AG11718, Accurate Biology) was used for PCR amplification and a real-time PCR machine (ABI-7500, ThermoFisher) was used to quantify the expression of mRNAs. β-actin was used as an endogenous control, and the expression levels were quantified using the 2^−ΔΔCt^ method. All primer sequences are listed in Additional file 2: Table S9, and each primer was detected in triplicate.

### Western blotting

Cells and tissues were lysed by RIPA buffer (P0013C, Beyotime Biotechnology) with protease and phosphatase inhibitor cocktail (P002, NCM Biotech), and total protein concentration was measured using a bicinchoninic acid protein assay kit (YSD-500 T, Yoche). Samples were denatured by boiling in 1 × loading buffer and run on a 10% SDS–PAGE gel. Membranes were incubated with primary antibodies overnight at 4 °C, washed three times with TBST, incubated with secondary antibodies for 1 h at room temperature, and developed using Ultra High Sensitive ECL Kit (G2020-25ML, Servicebio). The primary and secondary antibodies used and their dilution are listed in Additional file 2: Table S8. Western blots shown in the accompanying figures are derived from three independent experiments.

### Mouse studies

Mouse Studies Athymic nude mice (BALB/cA nu/nu) aged 4 to 5 weeks (Shanghai Jiao Tong University School of Medicine, Shanghai, China) were housed in sterile cages under laminar airflow hoods in a specific pathogen-free room at 22–25° with a 12-h light and 12-h dark schedule and fed with autoclaved chow and water ad libitum.

### In vivo subcutaneous xenograft

Female BALB/c nu/nu mice were purchased at 4 weeks of age and AtT20 cells were harvested and washed in PBS, resuspended with Hank's balanced salt solution (HBSS) about 1 × 10^6^ (per side/100 μL) concentrations were injected subcutaneously on the suitable sites per mouse. Treatment was started when the tumor sizes reached approximately 100 mm^3^; mice were randomized into different groups. An intraperitoneal injection of Activin A (5 mg/kg in 100 μl PBS) or PBS was administered every other day. Tumor volumes (per group) were measured with a digital caliper and calculated as length × width^2^ × 0.52. Relative tumor volume (RTV) was calculated by (RTV = TV_t_ / TV_0_, where TV_0_ is the tumor volume measured when starting drug treatment). The anti-tumor effect of drug treatment was calculated by drug-treated/control (T/C) ratio (T/C = RTV_treated_ / RTV_control_ × 100%). The mouse viscera were analyzed by HE staining to evaluate the safety of the inhibitor. The growth (Ki-67) and hormone (Pomc) of each tumor were measured by immunofluorescence (IF). The HE staining and IF analyses were performed, and the individuals performing the experiments were unaware of the samples being analyzed.

### Statistical analysis

All statistical analyses were performed using R 4.2.1. All heatmaps were generated by the R package pheatmap (https://CRAN.R-project.org/package=pheatmap). The *P*-values were two-sided. *P*-values of less than 0.05 were considered statistically significant. For adjustment of *P*-values, *P*-values were adjusted using the Benjamini and Hochberg method (alias false discovery rate, FDR).

## Results

### Single-cell transcriptional landscape of PitNETs

**Fig. 1:**
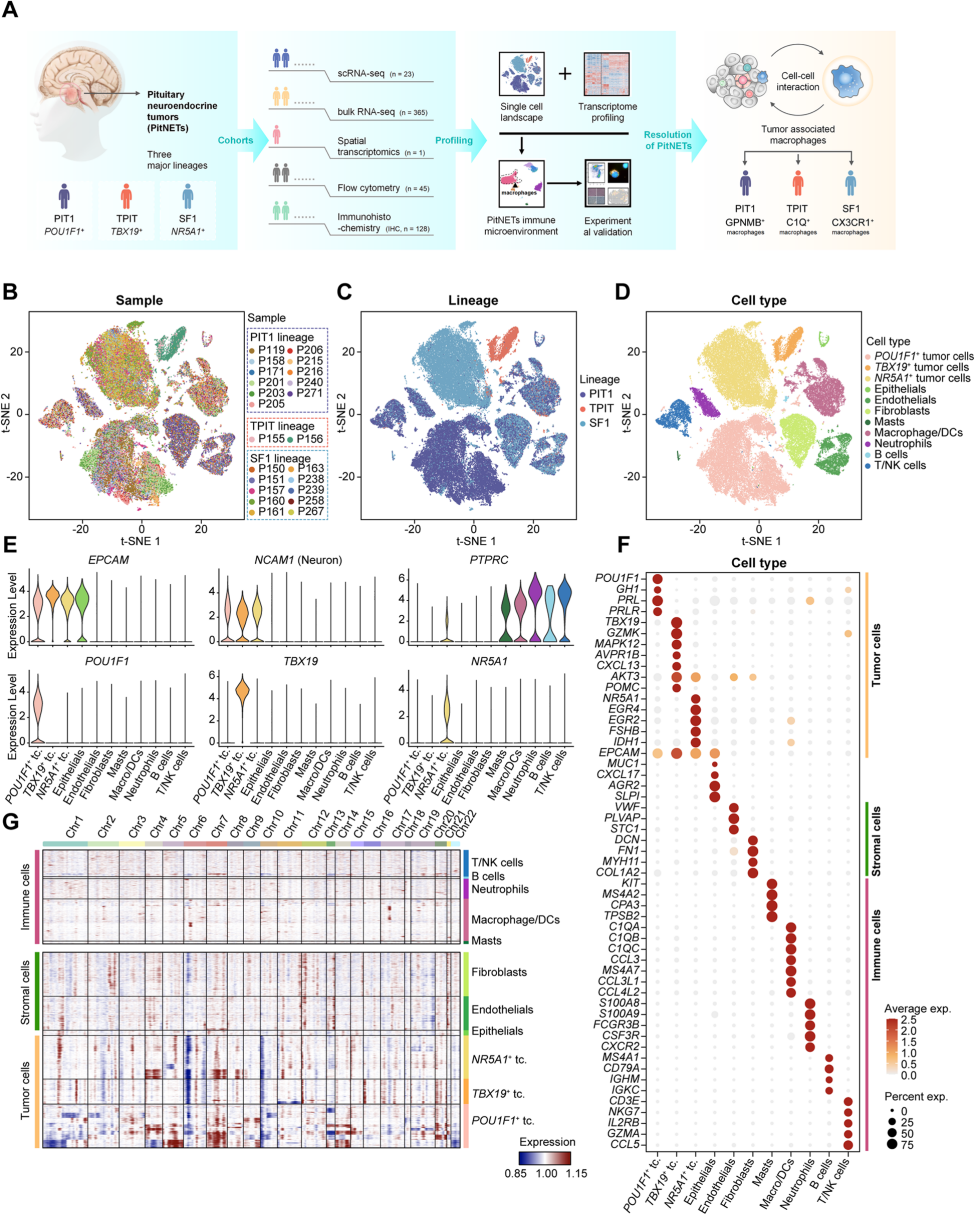
Single-cell analysis reveals the transcriptomic landscape in PitNETs. **A** Graphic overview of the study design and workflow. **B** Uniform Manifold Approximation and Projection (UMAP) map visualizes 69,539 cells, with color-coding representing individual samples. **C** UMAP overview color-coded by the three lineages observed. **D** UMAP overview color-coded by the identified cell types. **E** Violin plots depict marker gene expression levels across three types of tumor cells and immune cells. **F** Dot plot illustrating the specific marker genes associated with the 11 major cell types. **G** Heatmap displaying the chromosomal map of single-cell large-scale copy number variations (CNVs) inferred through scRNA-seq. Immune cells were used as a reference, and amplifications (red) or deletions (blue) were inferred by averaging expression over 100-gene stretches on each chromosome

In order to analyze the distribution of tumor cells and the cells in the tumor microenvironment (TME), we performed single-cell RNA sequencing (scRNA-seq) on the tumor tissues of 23 patients with PitNET. These tumors were classified into 11 PIT1 tumors, 2 silent TPIT tumors, and 10 SF1 tumors based on the expression levels of three transcription factors (Fig. 1A). After stringent quality control, we obtained 69,539 cells for further analysis. The batch effect of the scRNA-seq data across 23 samples was corrected with the Harmony algorithm [39, 50]. Subsequently, a total of 11 prominent cell types were discerned. Canonical markers were used to identify each cell type, and their visualization was performed using t-distributed stochastic neighbor embedding (t-SNE) (Fig. 1B–D) [39]. Despite the tumor cells which were annotated based on lineage-specific transcription factors, namely *POU1F1*^+^, *TBX19*^+^, and *NR5A1*^+^ tumor cells, every cell type appeared across the three lineages (Fig. 1D, Additional File 1: Fig. S1A-C). Besides lineage-specific transcription factors, tumor cell clusters shared expression of epithelial marker gene *EPCAM* and neuron marker gene *NCAM1* (Fig. 1E). The transmembrane protein tyrosine phosphatase gene *PTPRC (CD45)* is expressed in immune cells. We identified five subtypes of immune cells, including macrophages and DCs expressing *C1QA* and *MS4A7*, neutrophils expressing *S100A8* and *FCGR3B*, B cells expressing *MS4A1* and *CD79A*, T/NK cells expressing *CD3E* and *NKG7*, and mast cells expressing *KIT* and *MS4A2*. Two subtypes of stromal cells were identified: endothelial cells expressing *VWF* and *PLVAP* and fibroblasts expressing *COL1A2* and *FN1* (Fig. 1F). To further validate our findings, flow cytometry analysis was conducted on 45 PitNET samples. The results illustrated that macrophages exhibited the highest expression among CD45^+^ cells, while epithelial cells dominated the expression among CD45^−^ cells (Additional file 1: Fig. S1D).

Tumor heterogeneity can be characterized based on genetic and copy number variations (CNVs). Most PitNET are benign tumors whose subtypes depend on lineage-specific transcription factors [2]. The presence of CNVs in benign tumors and their impact on tumor control remains undisclosed. In order to examine the differences in CNVs among various lineages of PitNET, we measured the CNVs using the inferCNV method [50]. We found that the three lineages of PitNET had more frequent CNV events in tumor cells, suggesting that PitNETs also have many CNV events. PIT1 lineage harbored the highest number of CNVs (Fig. 1G).

We screened differentially expressed genes (DEGs) in three lineages of tumor cells using the FindMarkers function and performed functional enrichment analysis. The results reveal the most significantly up-regulated enriched signal pathways (Additional file 1: Fig. 2A-C). Notably, the *POU1F1*^+^ tumor cells exhibited the upregulation of genes involved in intracellular protein trafficking and extracellular protein secretion. The up-regulated DEGs in *TBX19*^+^ tumor cells were related to apoptotic signaling pathways and vesicle-mediated transport. *NR5A1*^+^ tumor cells showed the upregulation of genes associated with response to extracellular stimuli and regulation of neuronal death.

**Fig. 2:**
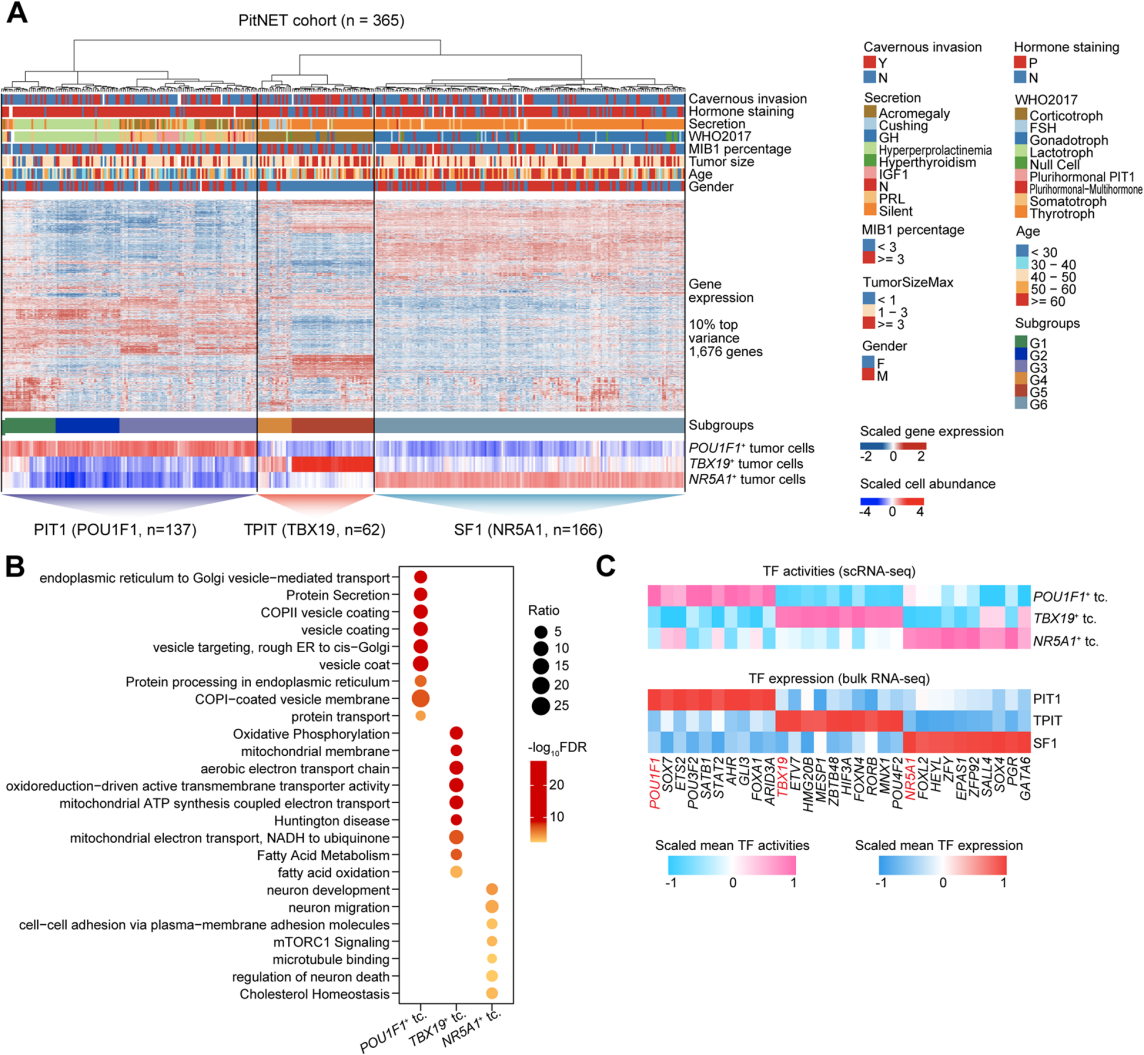
Tumor cells in PitNETs can be divided into three lineages based on their gene expression profile. **A** Gene expression patterns and clinical features of three distinct lineages in PitNETs. The columns represent PitNET patients, and the rows indicate gene expression levels or clinical features. **B** Bubble diagram illustrating the Gene Ontology (GO) biological processes (BP) associated with the three distinct tumor lineages. **C** Heatmap displaying the activation level of TF and their expression in the three PitNET lineages, as determined by scRNA-seq and bulk RNA-seq methods

### Distinct function modules of PitNETs subtypes

Our analysis of gene expression patterns at single-cell levels showed that the tumor cells derived from the PIT1, TPIT, and SF1 lineages of PitNETs have distinct tumor heterogeneity (Fig. 1C–D, and G). In addition, we previously classified six groups (G1 to G6) for PitNET patients based on bulk RNA-seq (*n* = 180), demonstrating the heterogeneity of PitNETs [51]. To further explore the heterogeneity of PitNET from the tissue and single-cell level, we integrated scRNA-seq with our in-house extended bulk RNA-seq (*n* = 365) to distinguish the PIT1, TPIT, and SF1 lineages of PitNETs, corresponding to G1–G3, G4–5, and G6, respectively (Fig. 2A). The classification shed light on a more specific clinical treatment. In the PIT1 lineage, the characterized genes were *PRL*, *PRLR*, *DLK1*, *ARHGAP36*, *PLCXD3*, *GH1*, *GHRHR,* and the transcription-factor-coding *POU1F1* (Additional file 1: Fig. S3A). The TPIT lineage had *TBX19*, *POMC*, *GZMK*, SST, *SOX3*, *AVPR1B*, *CARTPT*, and *CALB1* genes as feature genes (Additional file 1: Fig. S3B) [6, 51, 52]. Many of the characteristic genes in both PIT1 and TPIT lineages regulate the synthesis and secretion of hormones. On the other hand, the highly expressed genes in the SF1 lineage were *NR5A1*, *GATA2*, *IDH1*, *EGR2*-4, and *NSG2* (Additional file 1: Fig. S3C). These genes primarily participate in tumor development, differentiation, proliferation, and growth. More importantly, the characteristic transcription factor expression levels were coherent between scRNA-seq and bulk RNA-seq data (Fig. 2B). Furthermore, Gene Ontology (GO) enrichment analysis of DEGs for the enriched signal pathway identified key differences among the three lineages of PitNETs cells (Fig. 2C). The PIT1 lineage tumor cells were more involved in glycoprotein metabolic and biosynthetic processes; the TPIT lineage was involved in the transport and exocytosis of ions; and SF1 lineage was involved in gland development and synapse-related process (Fig. 2C). Collectively, both scRNA-seq and our expanded bulk RNA-seq data validated three distinct function modules of PitNETs.

### Three distinct subtypes of TIME associated with PitNETs lineages

**Fig. 3:**
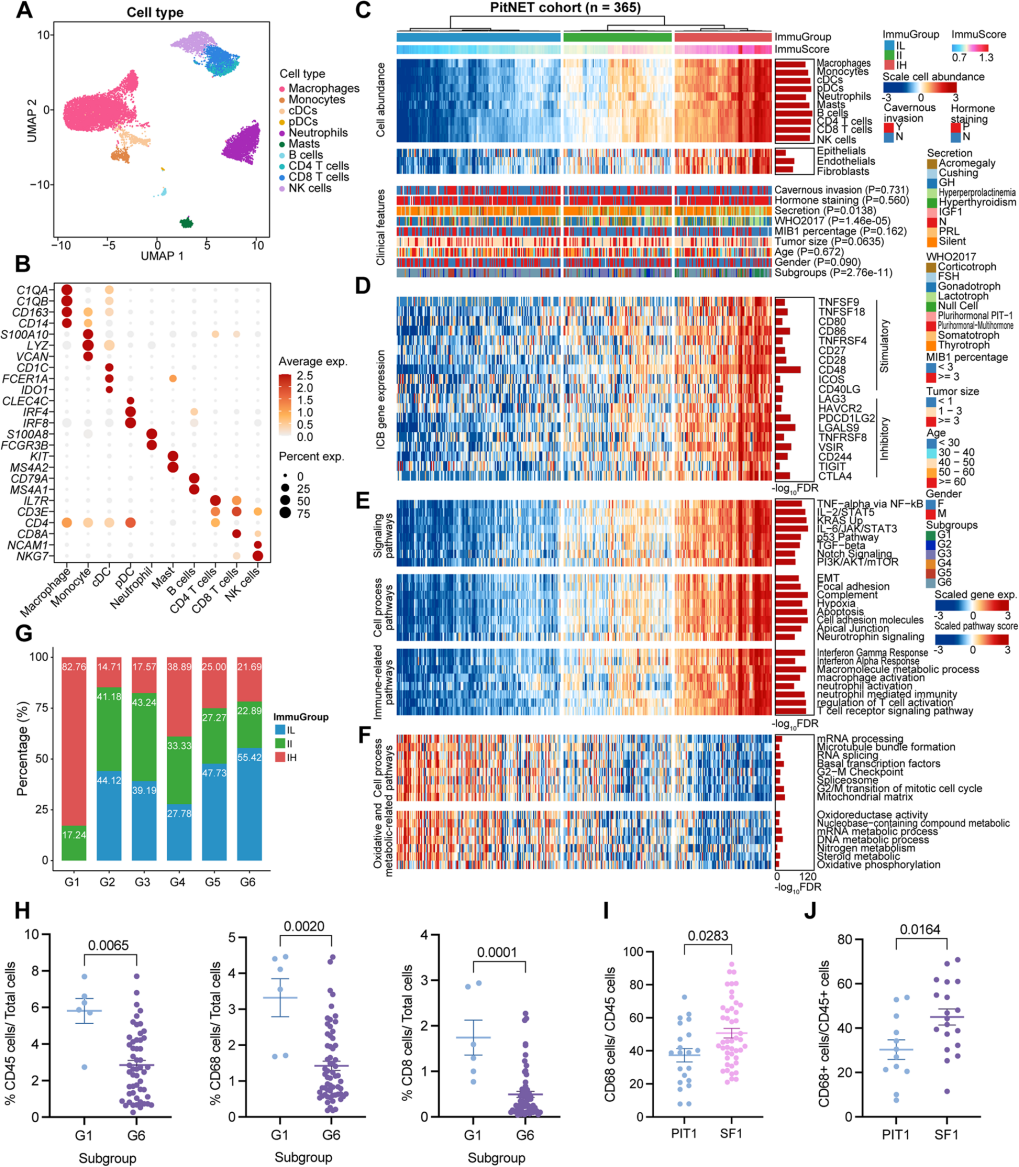
Profiling immune cell populations in PitNET. **A** Uniform Manifold Approximation and Projection (UMAP) map displaying 12,079 immune cells, with color-coding indicating individual cell types. **B** Dot plot illustrating the specific marker genes associated with the 10 major immune cell types. **C–F** The unsupervised clustering of PitNET RNA-seq cohorts based on the abundance of immune cells revealed three distinct TIME subtypes. The plot shows the abundance of immune cells in each subtype (**C**). Expression levels of immune checkpoint genes in the PitNET TIME subtypes (**D**). The pathways are enriched based on the DEGs (**E, F**). *P*-values were calculated using ANOVA and adjusted using FDR. **G** The composition of immune classification in samples from groups G1 to G6. **H** Bar plot indicating the proportion of positive cells stained for CD45, CD68, and CD8 in PitNET Tissue Microarray (TMA) using IHC with G1 (*n* = 6) and G6 (*n* = 52). *P*-values were calculated using the Wilcoxon rank-sum test. Results are presented as mean ± standard error of the mean in bar plots. **I–J.** Bar plot displaying the proportion of CD68/CD45 positive cells stained in PitNETs TMA using IHC with PIT1 (*n* = 21) and SF1 (*n* = 45) (**I**) and flow cytometry with PIT1 (*n* = 12) and SF1 (*n* = 19) (**J**). *P*-values were calculated using the Wilcoxon rank-sum test. Results are presented as mean ± standard error of the mean in bar plots

In addition to specific tumor cell composition across PitNETs lineages, we also found significant differences among the microenvironment. The highest abundance of stromal cells was found in SF1, and the most immune cell infiltration was seen in PIT1 compared to the other two subtypes (Additional file 1: Fig. S3D). However, the specific biological composition and functions of immune cells in the microenvironment of PitNET remain uncertain [2, 53]. Here, we drafted an immune landscape of PitNETs to explore the functional roles of TIME in different lineages. Based on distinct characteristic marker genes, we divided the 12,079 cells into 10 groups from cell clusters that were annotated to be immune cells, including macrophages, monocytes, dendritic cells (pDC and cDC), neutrophils, mast cells, B cells, T cells (*CD4*^+^T and *CD8*^+^T), and NK cells (Fig. 3A-B, Additional file 1: Fig. S4A-B). Notably, macrophages had *C1QA*, C1QB, and *CD163* as feature genes, while highly expressed *S100A10*, *LYZ*, and *VCAN* were observed in the monocyte cluster. Although each immune cell subset was present in most samples, we observed significant differences in their proportions in different lineages (Additional file 1: Fig. S4C-D). CD4^+^ T cells, CD8^+^ T cells, and NK cells were enriched in PIT1; mast cells were increased in SF1; neutrophils were enriched in both PIT1 and SF1, while macrophages showed the highest infiltration in TPIT.

To further investigate the TIME differences among PitNET lineages, we performed unsupervised clustering of PitNETs RNA-seq cohorts based on immune cell infiltration, identifying three distinct TIME subtypes (Additional file 2: Table S10), namely the “Immune Low” (IL), “Immune Intermediate” (II), and “Immune High” (IH) groups (Fig. 3C). The degree of immune cells infiltration increases progressively from group IL to IH (Additional file 1: Fig. S5A). Immune checkpoints are essential in immune responses and are critical as immunotherapy targets [54]. Either inhibitory (e.g., *LAG3*, *HAVCR2*) or stimulatory checkpoints (e.g., *TNFSF9*, *TNFNR5A18*) rose steadily from group IL group to IH, suggesting a co-regulation of stimulatory and inhibitory immune signaling pathways to maintain TIME equilibrium among different PitNET lineages. Furthermore, single-sample Gene Set Enrichment Analysis (ssGSEA) highlighted distinct signaling pathways enriched in the IH group (e.g., NFKB, PI3K-AKT-mTOR, NOTCH, WNT, P53), as well as cellular processes (e.g., EMT, hypoxia, and apoptosis). Moreover, the IH group exhibited immune-related enrichment of IFN-γ response, IFN-α response, and macrophage infiltration, indicating higher immune activity and immune cell infiltration in these tumors. These enriched pathways regulate cellular functions, including growth, metabolism, survival, differentiation, epithelial-mesenchymal transition, and apoptosis.

Furthermore, we found that the IH group accounted for 82.76% of G1 samples, while 55.42% of G6 samples belonged to the IL group (Fig. 3G). However, the three PitNET lineages showed different proportions of immune infiltration (Additional file 1: Fig. S5B). Further, we conducted TMA-IHC analysis, validating that the G1 subtype has significantly higher infiltration of immune cells, including CD45^+^ cells, CD68^+^ cells, and CD8^+^ T cells, compared to the G6 subtype (Fig. 3H). Intriguingly, compared to the G1 subtype, the SF1 lineage (G6), primarily characterized as “IL” tumors, displayed increased infiltration of TAMs (Fig. 3I–J). These findings indicate that PitNETs can be categorized into three TIME subtypes. The IH group was predominantly observed in the G1 subtype, while the IL group was primarily observed in the G6 subtype with a higher infiltration of macrophages.

### Distinct subtypes of tumor-associated macrophage in different PitNETs lineages

**Fig. 4:**
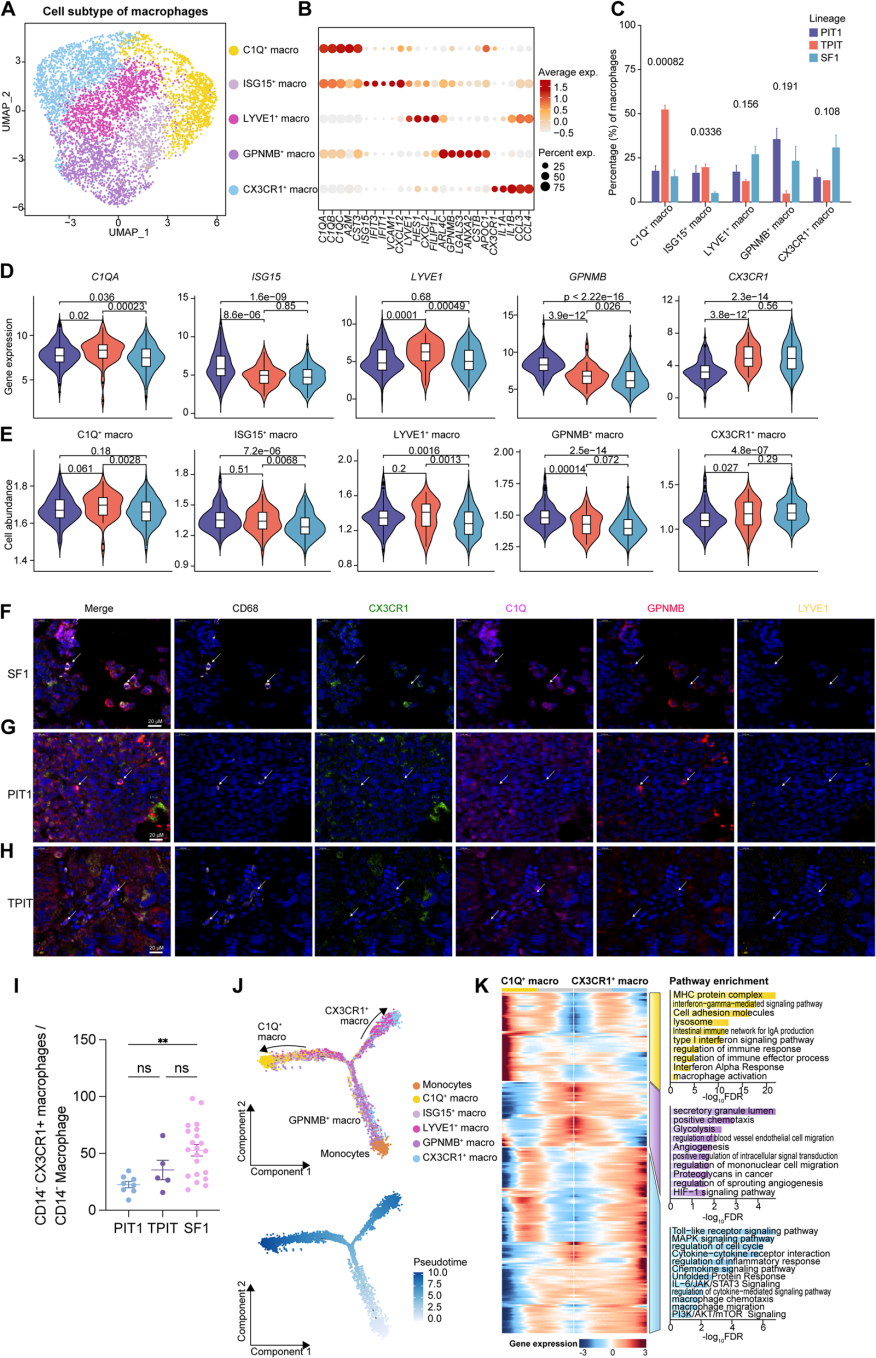
Three distinct subtypes of tumor-associated macrophage in three different PitNETs lineages. **A** UMAP map depicting macrophage cells color-coded by subtypes. **B** Dot plot displaying the specific marker genes for the five subtypes of macrophages. **C** The percentage of the five subtypes of macrophages observed in the three lineages. *P*-values are calculated using ANOVA. **D–E.** Violin plots show marker genes (**D**) expression levels and cell abundance (**E**) for the five subtype macrophages of three lineages. *P*-values were calculated using the Wilcoxon rank-sum test. **F–H.** Immunofluorescence (IF) image demonstrating the cell distribution of *CX3CR1*^+^ (**F**), *GPNMB*^+^ (**G**), and C1Q^+^ (**H**) macrophages in the three lineage tumor cells. **I**. Flow cytometry data presents the proportion of *CX3CR1*^+^ macrophages in the population across the three lineages. *P*-values were calculated using the Wilcoxon rank-sum test. Results are presented as mean ± standard error of the mean in bar plots. **J** Trajectory plot of monocytes and the five subtypes of macrophages in a two-dimensional state-space inferred by Monocle 2 analysis (up) and the transition trajectories along pseudotime (down). **K** Three-phase distribution of *C1Q*^+^ macrophages and *CX3CR1*^+^ macrophages along pseudotime. Heatmap depicting the dynamic expression changes of genes and related pathways of *C1Q*^+^ macrophages and *CX3CR1*^+^ macrophages along pseudotime

Our scRNA-seq data showed macrophages were the major immune cell population in PitNET tissue (Fig. 1D), and distinctive infiltration was seen in different PitNET lineages (Fig. 3I–J). To explore the functions of macrophages, we clustered the 5708 macrophages from 23 patients and identified 5 subclusters, including *C1Q*^+^, *ISG15*^+^, *LYVE1*^+^, *GPNMB*^+^, and *CX3CR1*^+^macropahges (Fig. 4A and Additional file 1: Fig. S6A-B). These macrophage subtypes had diverse distributions across the three PitNET lineages (Fig. 4C–D and Additional file 1: Fig. S6C-D); *C1Q*^+^ macrophages were mainly enriched in TPIT lineage tumors; *GPNMB*^+^ macrophages were enriched in PIT1 lineage tumors, and *CX3CR1*^+^ macrophages were enriched in SF1 lineage tumors. The distribution of macrophage subtypes in different PitNET lineages was validated using in-house bulk RNA-seq data based on a signature score of the top macrophage subtype-specifically expressed genes (Fig. 4E, Additional file 2: Table S11). We further examined the localization of *C1Q*^+^, *GPNMB*^+^, and *CX3CR1*^+^ macrophages in TPIT, PIT1, and SF1 lineages, respectively. Our multiple immunofluorescence labeling analysis provided strong evidence of the co-localization of *CX3CR1*^+^ macrophages and CD68 in SF1 lineage (Fig. 4F). Similarly, *GPNMB*^+^ macrophages were mainly co-localized with CD68 in PIT1 lineage (Fig. 4G). In contrast, *C1Q*^+^ macrophages were mostly co-localized with CD68 in the TPIT lineage (Fig. 4H).

Furthermore, trajectory analysis using three different methods witnessed a developmental path from monocytes to *LYVE*^+^ and *GPNMB*^+^ macrophages and finally to *CX3CR1*^+^ and *C1Q*^+^ macrophages (Fig. 4J, and Additional file 1: Fig. S7). Gene Ontology (GO) enrichment analysis on the genes enriched in each state revealed the different functions of macrophage subtypes. Furthermore, we performed KEGG pathway enrichment analysis to uncover distinct functions of five macrophage subtypes (Additional file 1: Fig. S8). The ribosomal signaling pathway supporting protein synthesis was enriched in TPIT-lineage-associated *C1Q*^+^ macrophages and thus might help to maintain elevated hormone secretion. The oxidative phosphorylation pathway providing energy and materials was enriched in POU1F1-lineage-associated *GPNMB*^+^ macrophages. The pathway of negative regulation for transforming growth factor beta (TGF-β) was enriched in SF1-lineage-associated *CX3CR1*^+^ macrophages and helped control the tumor growth. *C1Q*^+^ macrophages (characterized by the *C1QA/B/C* gene) showed a transitional phenotype from the initiate state to the immune-stimulated state, involving the upregulation of *HLA* genes and stimulation of the interferon signal pathway (Fig. 4K). The two clusters of genes exhibiting high expressions in *CX3CR1*^+^ macrophages play a critical role in immune response and cytokine–cytokine receptor interaction.

### Different regulation of macrophage-tumor cell interaction axis in PitNETs

**Fig. 5:**
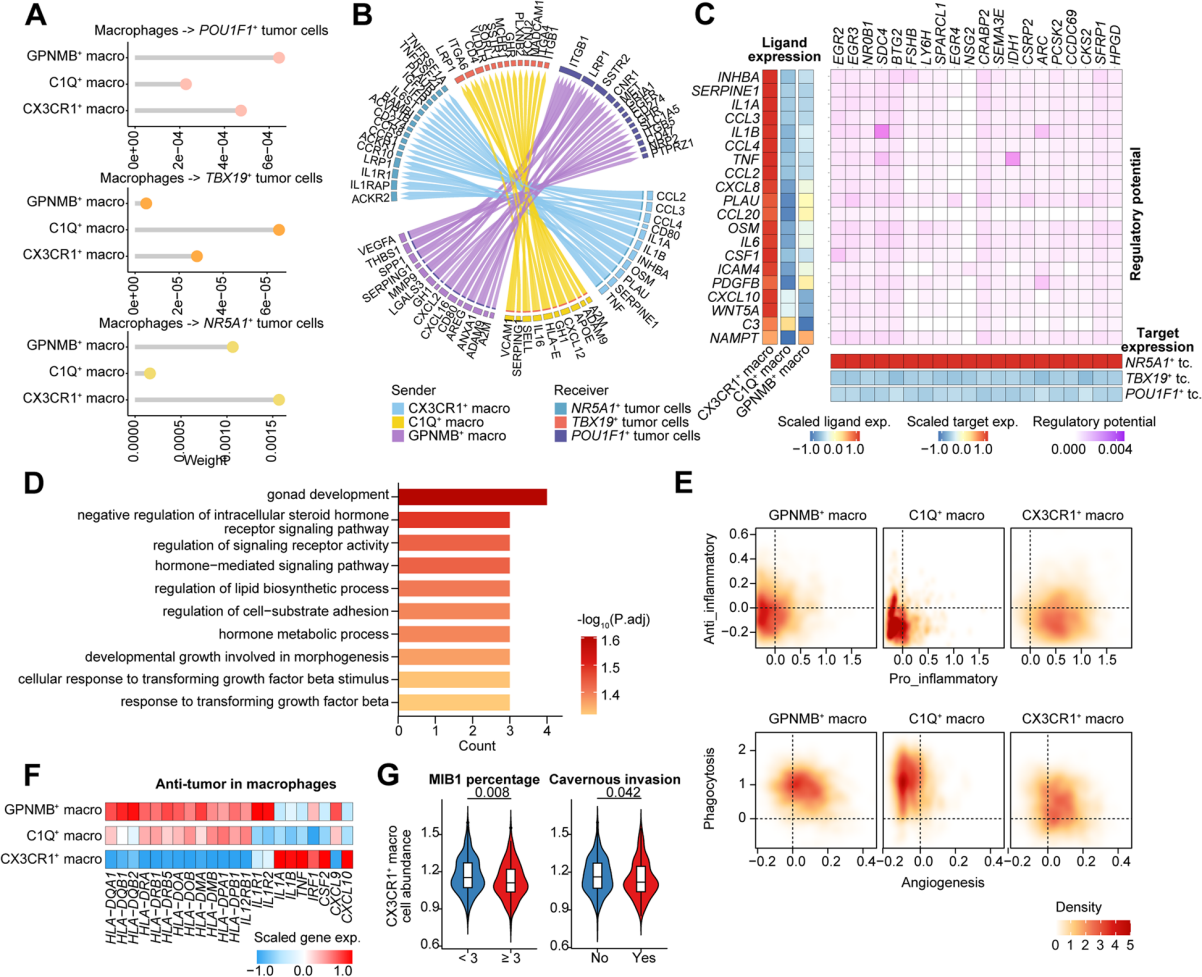
*CX3CR1*^+^ macrophages interact with *NR5*A1^+^ tumor cells. **A** Interaction weights between TAM subpopulations and the three lineages of tumor cells. **B** Ligands and receptor pairs are involved in the interaction between TAM subpopulations and the three lineages of tumor cells. **C** Heatmap displaying the expression levels of ligands highly expressed in *CX3CR1*^+^ macrophages (left) and the expression levels of corresponding target genes on *NR5A1*^+^ tumor cells (right). **D** Signal pathways enriched from the ligand–receptor interaction between *CX3CR1*^+^ macrophages and *NR5A1*^+^ tumor cells. **E** Evaluation of the anti/pro-inflammatory and angiogenesis/phagocytosis functions of the three subtypes of macrophages. **F** Heatmap representing the mean expression levels of anti-tumor genes in the three subtypes of macrophages. **G** Violin plots illustrating the cell abundance of *CX3CR1*^+^ macrophages in samples categorized as low (< 3) or high (≥ 3) MIB1 percentage (on the left), as well as in samples with or without invasion. *P*-values were calculated using the Wilcoxon rank-sum test

To understand the biology of macrophage–tumor cell interactions, we performed the CellChat analysis along the macrophage–tumor cell axis in different PitNET lineages. The results demonstrated that *CX3CR1*^+^, *C1Q*^+^, and *GPNMB*^+^ macrophages exhibited the highest interaction with *NR5A1*^+^, *TBX19*^+^, and *POU1F1*^+^ tumor cells, respectively (Fig. 5A). This highlighted distinct ligand–receptor pairs at each macrophage–tumor cell axis (Fig. 5B). To explore the mediators and downstream targets of the macrophage–tumor cell axis, we performed NichenetR analysis. We found that *CX3CR1*^+^ macrophages regulated *NR5A1*^+^ tumor cells through regulation of signaling receptor pathway and TGF-β stimulus (Fig. 5D). The target genes and cluster-enriched pathways of *C1Q*^+^ and *GPNMB*^+^ macrophages were associated with protein maturation, response to corticosteroid and JAK-STAT, T cell proliferation and activation, and response to cAMP functions (Additional file 1: Fig. S9A-D). Additionally, we investigated the functional profiles of the three macrophage clusters. *CX3CR1*^+^ macrophages exhibited higher activity in pro-inflammatory responses and angiogenesis. In contrast, *C1Q*^+^ macrophages demonstrated a high level of phagocytosis (Fig. 5E). Conversely, *GPNMB*^+^ macrophages were involved in angiogenesis and phagocytosis (Fig. 5E). Furthermore, *CX3CR1*^+^ macrophages displayed a pro-inflammatory profile, while *GPNMB*^+^ macrophages represented to be anti-inflammatory. *C1Q*^+^ macrophages exhibited minimal regulation of inflammation. Gene expression analysis within each macrophage cluster demonstrated that *CX3CR1*^+^ macrophages showed the upregulation of pro-inflammatory genes, including *IL1B*, *TNF*, *CXCL9*, and *CXCL10* (Fig. 5F). This suggested that *CX3CR1*^+^ macrophages contributed to the pro-inflammatory TIME of PitNETs. Furthermore, we observed a downregulation of *CX3CR1*^+^ macrophages in cases of MIB1-high and cavernous invasion PitNETs (Fig. 5G). Together, these findings suggested a potential association of reduced numbers of *CX3CR1*^+^ macrophages with the increased proliferation and aggressive characteristics of SF1 lineage PitNETs.

### The INHBA-ACVR1B axis promotes tumor cell apoptosis

**Fig. 6:**
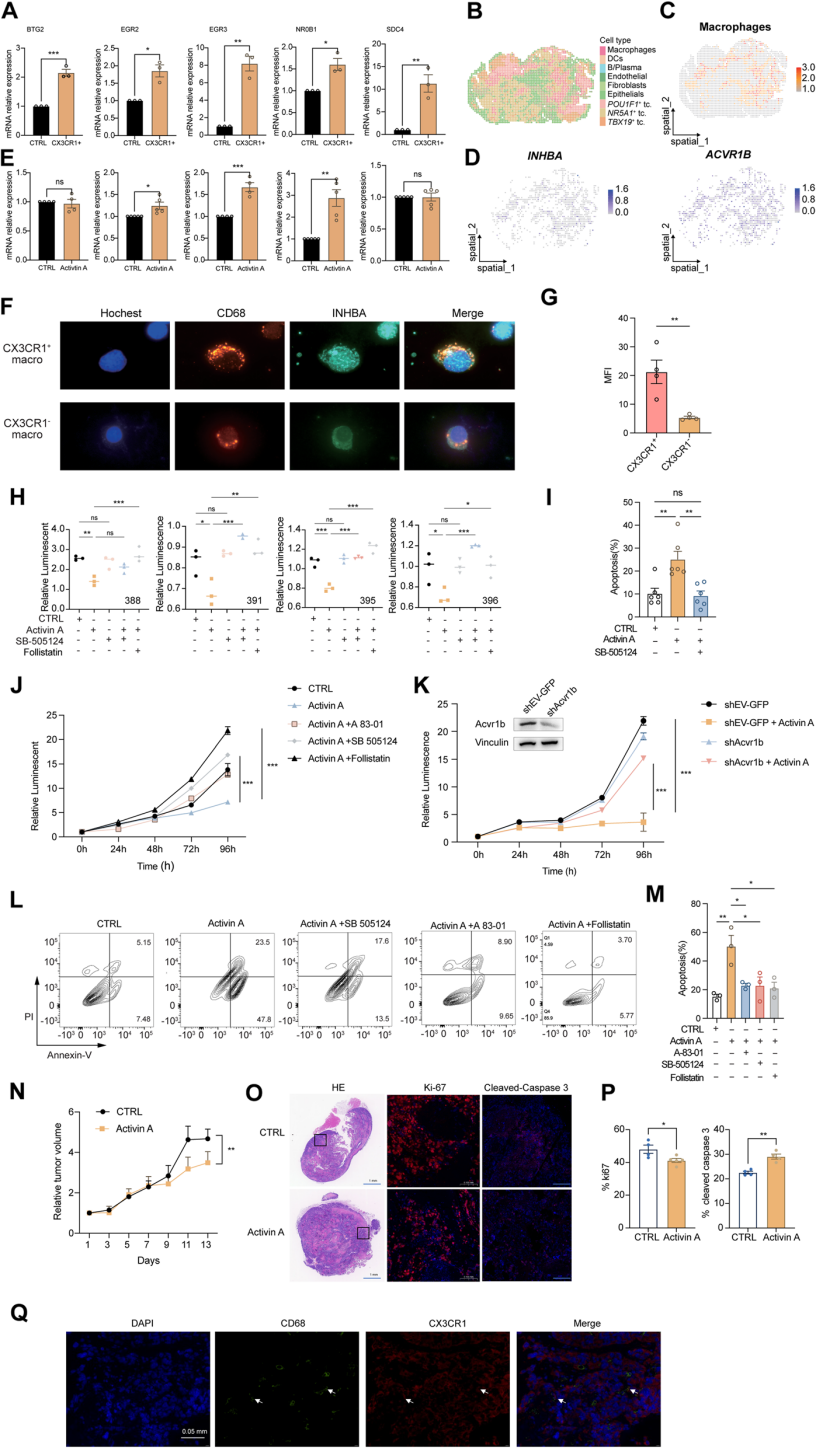
*CX3CR1*^+^ macrophages inhibit the growth of NR5A1 ^+^ tumor cells through INHBA/activin A—ACVR1B axis. **A** Histogram plot revealed the gene expression alteration of the tumor cells co-cultured with *CX3CR1*^+^ macrophages (*n* = 3). **B–D** HE images with pie charts in each spot colored by annotation showing the cellular composition in the ST sample (**B**). The spatial feature plot shows the location and proportions of macrophages in ST spots using deconvolution results (**C**). Spatial feature plots show the expression of INHBA and its receptor ACVR1B in the macrophage-containing spots (**D**). **E** Histogram plot revealed the gene expression alteration of the tumor cells co-cultured with activin A. **F–G** The representative image of immunofluorescence revealing the specific expression of INHBA in *CX3CR1*^+^ and *CX3CR1*^−^ macrophages (**F**) and the statistics of INHBA mean fluorescence intensity (MFI) in *CX3CR1*^+^ and *CX3CR1*^*−*^ macrophages in three patients (**G**). **H–I** The cell viability (**H**) and apoptosis (**I**) in the four NR5A1^+^ primary cell treated activin A (10 ng/ml) with or without SB-505124 (1 μM) or follistatin (100 ng/ml). The histogram shows the cells' apoptosis in different conditions for the primary cell (**I**). **J** Cell viability assessment in the AtT20 cell line treated with Activin A (10 ng/ml) in the presence or absence of SB-505124 (1 μM), A 83–01 (1 μM), or follistatin (100 ng/ml). **K** Cell viability analysis in AtT20 cell line with shEV-GFP or shAcvr1b treated with Activin A (10 ng/ml). **L–M.** Apoptosis assessment in the AtT20 cell line treated with Activin A (10 ng/ml) in the presence or absence of SB-505124 (1 μM), A 83–01 (1 μM), or follistatin (100 ng/ml). The histogram illustrates the apoptosis levels of cells under various conditions in the AtT20 cell line (**M**). **N–P** Tumor growth curve (**N**). The xenografts harboring mice were injected intraperitoneally with activin A release preparations, 5 mg/kg weight, every other day with PBS. Representative images of H&E and IF staining (Ki-67 and Cleaved-caspase 3) of resected AtT20 tumors from an experimental study between different groups (**O**). Scale bar as indicated. Statistical analysis of Ki67 and Cleaved-caspase 3 percentages in control and treated Att20 tumors (**P**). **Q** The staining revealed the co-localization of CD68 and CX3CR1 in xenografts harboring mice. Arrow indicated the CD68 and CX3CR1 positive staining. Scale bar as indicated. *P < 0.05, **P < 0.01, ***P < 0.001, ****P < 0.0001. *P*-values were calculated using the Wilcoxon rank-sum test. Results are presented as mean ± standard error of the mean in bar plots

To investigate the regulatory role of *CX3CR1*^+^ macrophages on *NR5A1*^+^ tumor cells, we co-cultured PitNETs isolated *CX3CR1*^+^ or *CX3CR1*^*−*^ macrophages with autologous *CD45*^−^ tumor cells. We found that *CX3CR1*^+^ macrophages enhanced the expression of the corresponding target tumor inhibitory genes, such as *BTG2, EGR2, ERG3, NR0B1*, and *SDC4,* on *NR5A1*^+^ tumor cells (Fig. 6A). INHBA-ACVR1B was found to be the top-ranked ligand-receptor pair among *CX3CR1*^+^ macrophages and *NR5A1*^+^ tumor cells interaction. Notably, *CX3CR1*^+^ macrophages enriched *NR5A1*^+^ tumor cells and INHBA-ACVR1B were locationalized closely in the spatial transcriptome of SF1 lineage tissue (Fig. 6B–D and Additional file 1: Fig. S10A). Furthermore, we found that INHBA protein treatment up-regulated three tumor inhibitory genes, namely EGR2, ERG3, and NR0B1 (Fig. 6E). Also, immunofluorescence analysis on flow-sorted *CX3CR1*^+^ macrophages validated the expression of INHBA in the *CX3CR1*^+^ macrophages population (Fig. 6F–G).

To confirm the role of INHBA on the CX3CR1^+ ^macrophages NR5A1^+^ tumor cells interaction axis, we treated primary cells and PitNET cell lines with INHBA. We found that INHBA inhibited cell viability in SF1 lineage primary cells (Fig. 6H) and induced apoptosis (Fig. 6I). We further used SB-505124, which reversed the inhibitory effect of INHBA (Fig. 6H and I). Similar outcomes were shown in the AtT20 cell line, where INHBA caused apoptosis and decreased cell proliferation. Both effects may be reversed by follistatin and reversible ATP competitors that are selective for ALK4 and ALK5 (SB-505124 and A 83–01) (Fig. 6J, L, and M). To further confirm the dependency of the inhibitory effect on the INHBA receptor Acvr1b, we conducted experiments demonstrating a significant reversal of the inhibitory effect upon downregulating Acvr1b using shRNA (Fig. 6K). However, no inhibitory effect of INHBA was observed in the MMQ and GH3 PitNET cell lines (Additional file 1: Fig. S10B). This can be attributed to the low expression of ACVR1B (Additional file 1: Fig. S10C). Moreover, in vivo drug treatment experiments were subsequently performed to confirm the validity of the above results. Tumor growth of AtT20 xenografts in a mouse subcutaneous model was reduced by continuous therapy with INHBA (activin A) (Fig. 6N). The hematoxylin and eosin (H&E) staining results confirmed that activin A treatment induced similar tumor necrosis in the AtT20 xenografts. However, the Ki-67 and Cleaved-caspase 3 staining results showed decreased proliferation and increased apoptosis in treatment with activin A (Fig. 6O and P). To confirm the expression of *CX3CR1*^+^ macrophages in AtT20 xenografts, immunofluorescence (IF) staining results confirmed the co-localization of CD68 and CX3CR1 in the tissue (Fig. 6Q). Consequently, these results propose that *CX3CR1*^+^ macrophages may trigger apoptosis in tumor cells through the INHBA-ACVR1B axis.

## Discussion

Different phenotypes of TIME, such as immune-inflamed microenvironment, immune-excluded microenvironment, and immune-desert microenvironment, have been associated with multiple cancer types and immunotherapy efficacy [21, 55]. In particular, many recent studies have revealed that different macrophage subtypes execute specific functions in tumors, and therefore, macrophage-targeted therapy is an attractive immunotherapy strategy. Despite the emerging studies [21, 56] on the components of tumor immune microenvironment (TIME), the TIME of PitNETs is not fully deciphered at the transcriptomic level, limiting the progress of clinical research on PitNETs immunotherapy. This study provides a comprehensive analysis of the immune landscape and macrophage heterogeneity in PitNETs, highlighting the potential roles of specific macrophage populations in tumor progression. We performed single-cell, bulk, and spatial RNA-seq analyses of different PitNET lineage samples to identify tumor heterogeneity and distinguish function modules of PitNET subtypes. Finally, three distinct TIME subtypes associated with PitNETs lineages were identified. TAM subtypes interact with different PitNET lineages; *CX3CR1*^+^ TAMs interact with *NR5A1*^+^ tumor cells through the INHBA-ACVR1B axis, promoting tumor cell apoptosis.

Our previous study used bulk RNA-seq to classify 180 PitNET patient samples from three lineages into 6 subtypes (G1 to G6), revealing the heterogeneity of the PitNET lineage [51]. Using proteomics, Zhang et al. also identified distinct clusters of PitNET heterogeneity showing different functional associations [7]. Here, we further highlighted the tumor heterogeneity and TIME of three transcription factor(TF)-based lineages in PitNETs from a single-cell perspective. Our results complement previous studies concerning the heterogeneity of different lineages in PitNETs [3, 5, 6]. We identified that different lineages of PitNET not only have distinct characteristics in TF-related transcriptomic profiles and functional modules but also exhibit genetic variation and CNVs. We screened differentially expressed genes (DEGs) in three types of tumor cells. Among these, the *POU1F1*^+^ tumor cells demonstrated intracellular protein trafficking and extracellular protein release. This aligned with the clinical manifestation of abnormally high secretion of certain hormones in the PIT1 lineage [5]. A recent study also supported these findings and demonstrated an inverse correlation between differentiation status and recurrence within the PIT1/TPIT lineage compared to the SF1 lineage [57]. In particular, we combined 23 single-cell and 365 in-house bulk RNA-seq to reveal that all 3 PitNET subtypes have distinct immune-infiltrated patterns. The G1 subtype (PIT1 lineage) exhibited the highest immune infiltration, while the G6 subtype (SF1 lineage) showed the lowest immune infiltration. Concisely, the molecular phenotypes of PitNETs can be characterized by the immune infiltration phenotypes, providing a novel insight into the clinical diagnosis of PitNETs. This also suggests that the functional differentiation between G1-3 (PIT1 lineage) and G4-5 (TPIT lineage) subgroups may be controlled by TIME.

The heterogeneity of macrophages endows them with diverse functions, such as phagocytosis, antigen presentation, cytokine production, and tissue remodeling [20]. Using scRNA-seq, previous studies identified multiple TAM subtypes with distinct gene signatures and functions [58, 59]. Earlier studies often determined the proportion of macrophages by employing pan markers such as CD68, CD163, or CD204. These indicators investigated possible relationships between treatment response and tumor progression [60]. M2 macrophage infiltration is the primary immunological landscape that the PitNETs subtypes exhibit [61]. The cell-to-cell interactions of pitNETs and macrophages indicate potential therapeutic adjuvant for PitNET treatment [27, 62]. However, current research does not always point to successful treatment outcomes when using classical immune checkpoints like PD-L1 CTLA4 as a target [55, 63]. Here, we identified five distinct macrophage clusters that were differentially distributed and interacted with tumors of three PitNET lineages. Our research indicates that the traditional method of classifying TAMs into distinct M1 and M2 subtypes may not be fully relevant when studying PitNETs. TAMs in PitNETs exhibit features consistent with M1 and M2 phenotypes, disregarding the simplistic dichotomy commonly used in macrophage classification [64]. However, according to a previous study, most TAMs expressed M2-related genes [61]. *CX3CR1*^+^ macrophages displayed the upregulation of pro-inflammatory properties and intense interaction with *NR5A1*^+^ tumor cells; *C1Q*^+^ macrophages showed increased phagocytosis activity and strong interaction with *TBX19*^+^ tumor cells; *GPNMB*^+^ macrophage with an intermediate level of angiogenesis and phagocytosis showed preferential interactions with P*OU1F1*^+^ tumor cells. Concisely, different macrophage subtypes in PitNET have different functions and drive differentiated macrophage–tumor interaction axis. Gaining insight into the unique functions of various macrophage subsets and discerning the disparities between different types of PitNETs enhance the development of therapeutic approaches for PitNETs. *CX3CR1*^+^ macrophages were reported to have both anti- or pro-tumor effects in malignant tumors, and therefore, their functions are controversial [65–68]. Here, we demonstrated that *CX3CR1*^+^ macrophages exhibit anti-tumor effects, displaying a pro-inflammatory phenotype.

Furthermore, the expression of anti-tumorigenic genes (ERG2, ERG3, and BTG2) was promoted by the interaction between *CX3CR1*^+^ macrophages and *NR5A1*^+^ tumor cells through the ligand-receptor INHBA-ACVR1B pair [69, 70]. This was in agreement with earlier research [71] that ACVR1B can restore activin antiproliferative effects in human pituitary tumor cells. Interleukin-1 (IL-1), interferon-γ (IFN-γ), and tumor necrosis factor-α (TNF-α) were among the pro-inflammatory cytokines that were reduced by blocking FKN-CX3CR1 interaction, suggesting that this pathway could be a target for the therapy of rheumatoid arthritis [69]. Treatments for our condition may be viewed in the opposite direction. Our findings suggest a potential inhibitory role of *CX3CR1*^+^ macrophages in tumor growth within the SF1 lineage of PitNETs. Clinically, INHBA agonists can promote the INHBA-ACVR1B pathway to eliminate SF1 lineage pituitary tumors or increase the infiltration of *CX3CR1*^+^ macrophages in other lineages, helping PitNET treatment.

Our current analysis is limited by the scarcity of secreting TPIT lineage tumors in the scRNAseq dataset, as only two silent TPIT available samples exist. The primary reason for this limitation is the low frequency of secreting TPIT-related cases, which makes it challenging to gather a large enough sample size for thorough research. Due to the infrequency of secreting TPIT lineage tumors, it is crucial to use caution when interpreting results related to this specific subtype. Furthermore, limitations in T cells analysis have been identified, characterized by inadequate data quantity and inconclusive grouping. To overcome these problems, we suggest enhancing the presence of T cells in sequencing data by enriching CD45 in future investigations. This strategic improvement will enable more accurate categorization of immune cells and thorough analysis of the functions of T cells, therefore enhancing our understanding of the immune response in the tumors under investigation.

**Fig. 7:**
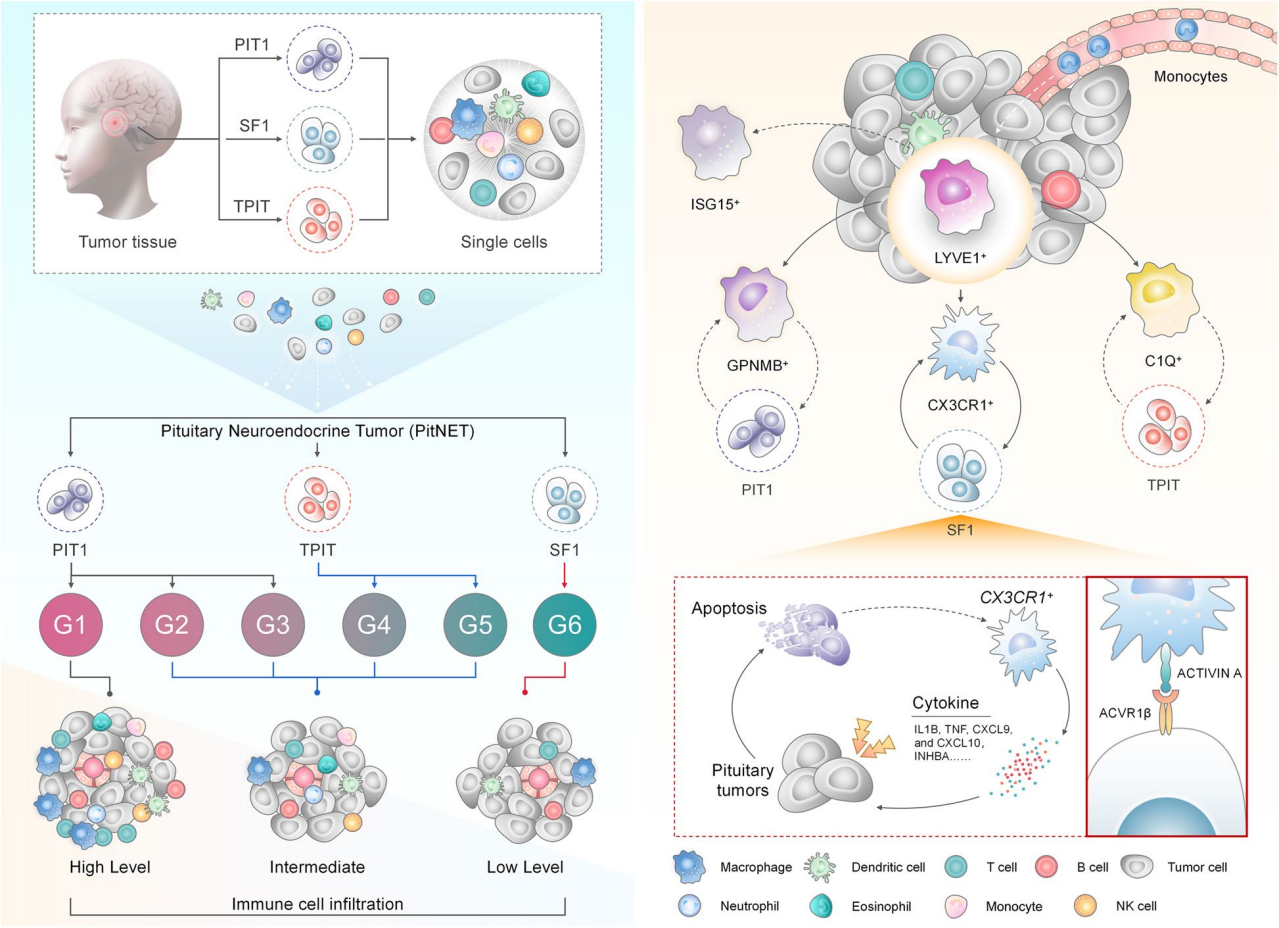
Working model of immune-infiltrating phenotypes and macrophage–tumor interaction axes in diverse lineages of pituitary neuroendocrine tumors

In summary, integrated analysis of bulk, single-cell, and spatial transcriptomic profiles on PitNET offered a comprehensive perspective on the diverse cellular compositions of PitNETs (Fig. 7). The identification of immune-infiltrating phenotypes, TAM subtypes, and distinct interaction axes of TAM-tumor cells provided valuable resources for future in-depth studies. Mainly, *CX3CR1*^+^ macrophages may hold significant promise in regulating tumor angiogenesis and inflammation. Here, our findings enable the categorization of molecular phenotypes of PitNETs according to the infiltration of immune cells and imply the possibility of treating PitNETs with macrophage subtype-based therapies. Additional investigation is required to elucidate the functions of different immune cells during the TIME of PitNETs.

## Conclusions

We conducted an in-depth exploration of the immune microenvironment within PitNETs, with a specific focus on the interactions between macrophages and tumor cells. Utilizing single-cell RNA sequencing analysis, our study unveiled three distinct subtypes of the TIME, each characterized by varying degrees of immune cell infiltration and macrophage activity. These findings underscore the significance of considering the tumor microenvironment in the therapeutic approach to PitNETs, particularly highlighting potential strategies targeting TAMs. Moreover, our research identified specific axes of interaction between macrophages and tumor cells, which may serve as novel targets for therapeutic intervention. The identification of these interaction axes not only enhances our understanding of the complexity of the immune microenvironment in PitNETs but also provides valuable insights for future clinical interventions. These discoveries hold promise for stimulating further research aimed at uncovering the potential of immunotherapy in treating PitNETs.

### Supplementary Information

**Additional file 1.** **Figure S1-S11. **All supplementary figures.

**Additional file 2. Table S1-5**. Clinical information of Bulk-RNA seq (Table S1), scRNA-seq (Table S2), TMA (Table S3), FC (Table S4) and clinical information for experiments (Table S5).** Table S6**. Quality control and filtration. **Table S7.** Spatial feature expression plots of ACVR1B coexpressing with INHBA and in adjacent spot. **Table S8**. List of antibodies and chemical dyes. **Table S9**. qPCR Oligonucleotide Sequences.** Table S10.** Cell type-specific markers in the UMAP representation of PitNETs.** Table S11.** Specific markers of macrophages.

## Data Availability

The raw sequence data reported in this paper have been deposited in the Genome Sequence Archive in National Genomics Data Center, China National Center for Bioinformation/Beijing Institute of Genomics, Chinese Academy of Sciences (GSA-Human: HRA005096) that are publicly accessible at https://ngdc.cncb.ac.cn/search/specific?db=hra&q=HRA005096 [70]. Code used in the paper is available on GitHub (https://github.com/NRCTM-bioinfo/PitNETs_scell_proj) [71] or FigureShare (10.6084/m9.fgshare.25439944) [72].
