# Supplementary material for: Single-cell transcriptomics reveal distinct immune-infiltrating phenotypes and macrophage–tumor interaction axes among different lineages of pituitary neuroendocrine tumors
Source: Genome Med. 2024 Apr 24;16:60. doi: 10.1186/s13073-024-01325-4 (PMC11040908; doi:10.1186/s13073-024-01325-4)
Supplement: Supplementary file 1 — Additional file 1. Figure S1-S11. All supplementary figures. [file 13073_2024_1325_MOESM1_ESM.docx]

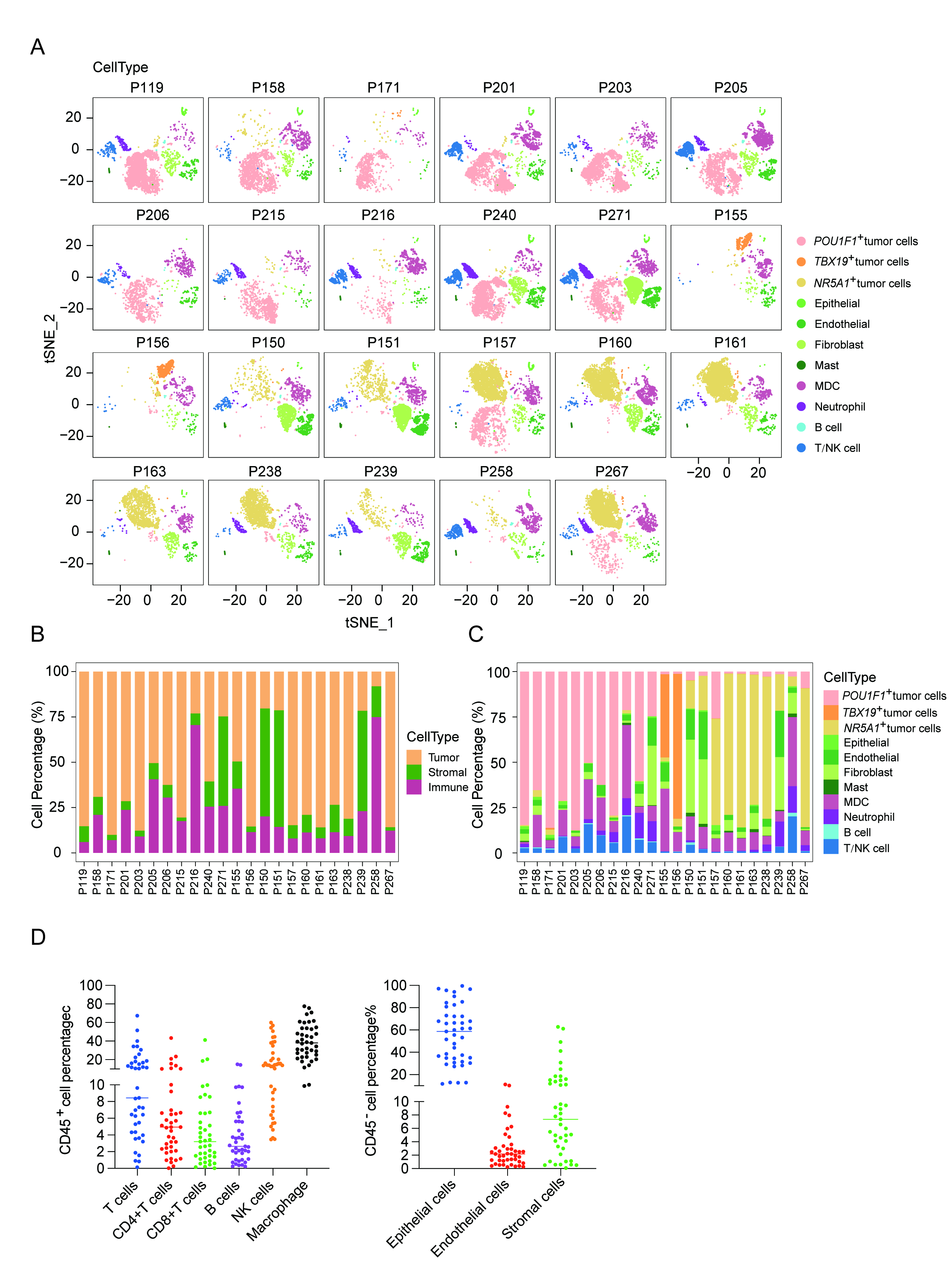


**Figure S1**. **A-C.** UMAP map of the cells for each sample color-coded by 11 cell types (**A**). The cell percentages of the three main cell types (**B**) and 11 cell types (**C**) for each sample, **D**, FC data showed the cell proportions of the CD45^+^ and CD45^-^ cells.


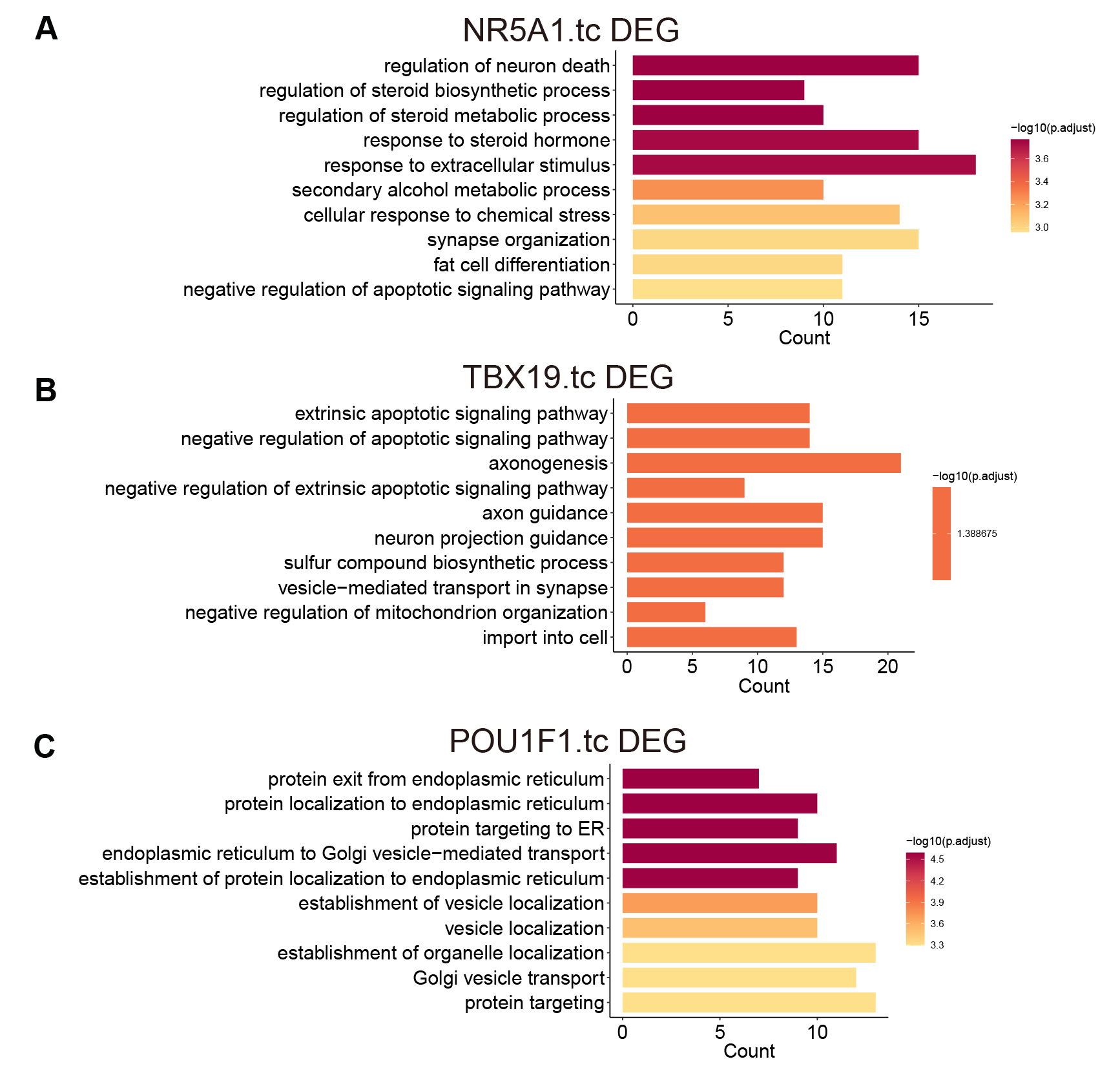


**Figure S2. A-C.** Function enrichment of the CNV region of the three lineages of tumor cells: *NR5A1^+^* tumor cells (**A**), *TBX19^+^* tumor cells (**B**), and *POU1F1^+^* tumor cells (**C**).


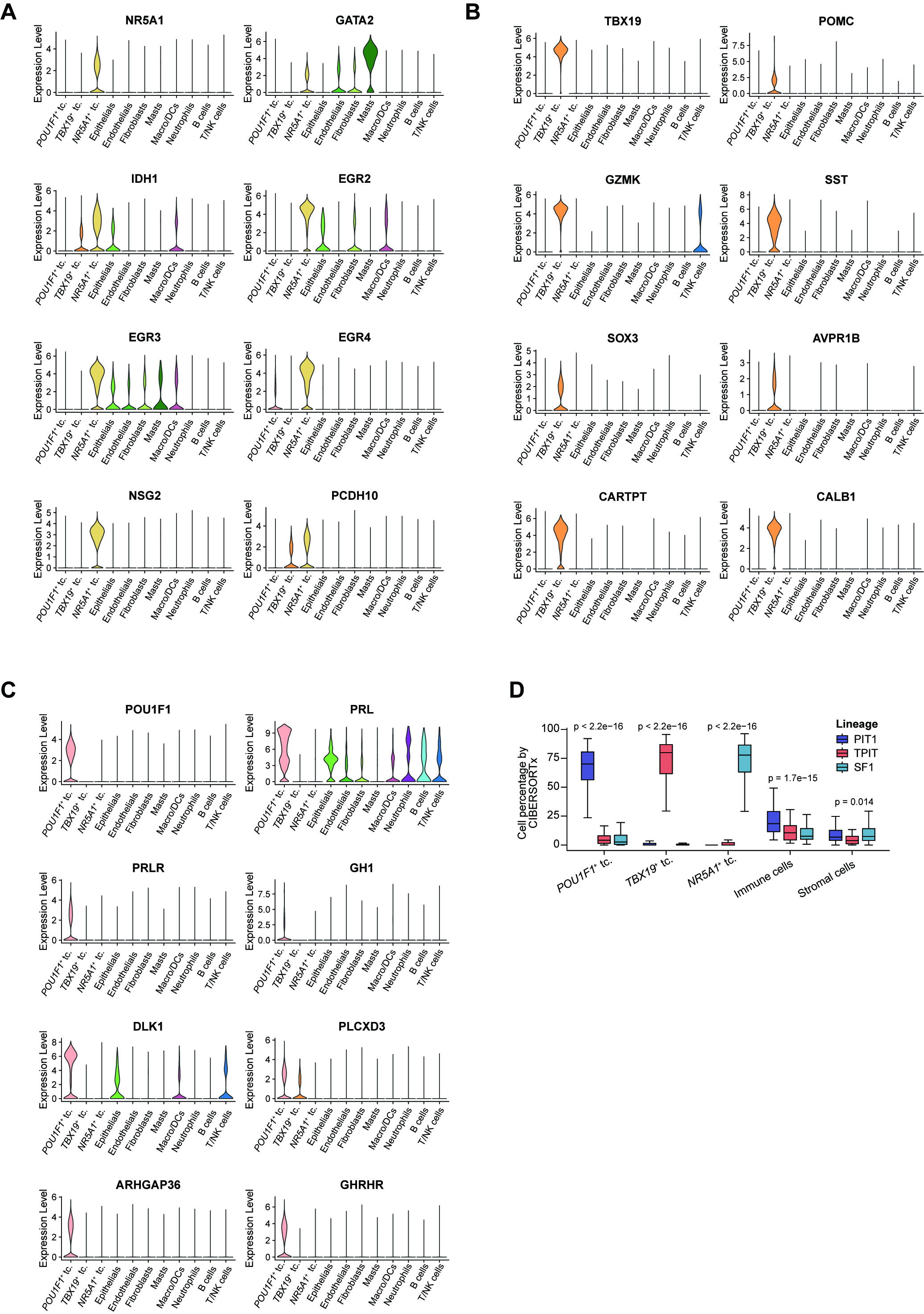


**Figure S3.** **A-C.** Violin plots visually represent the expression levels of marker genes across three types of tumor cells: *POU1F1^+^* tumor cells (**A**), *TBX19^+^* tumor cells (**B**), and *NR5A1^+^* tumor cells (**C**). **D.** The histogram shows the proportions of the 5 cell types in three lineages, including the *POU1F1*^+^ tumor cells, *TBX19*+ tumor cells, *NR5A1*^+^ tumor cells, immune cells, and stromal cells.


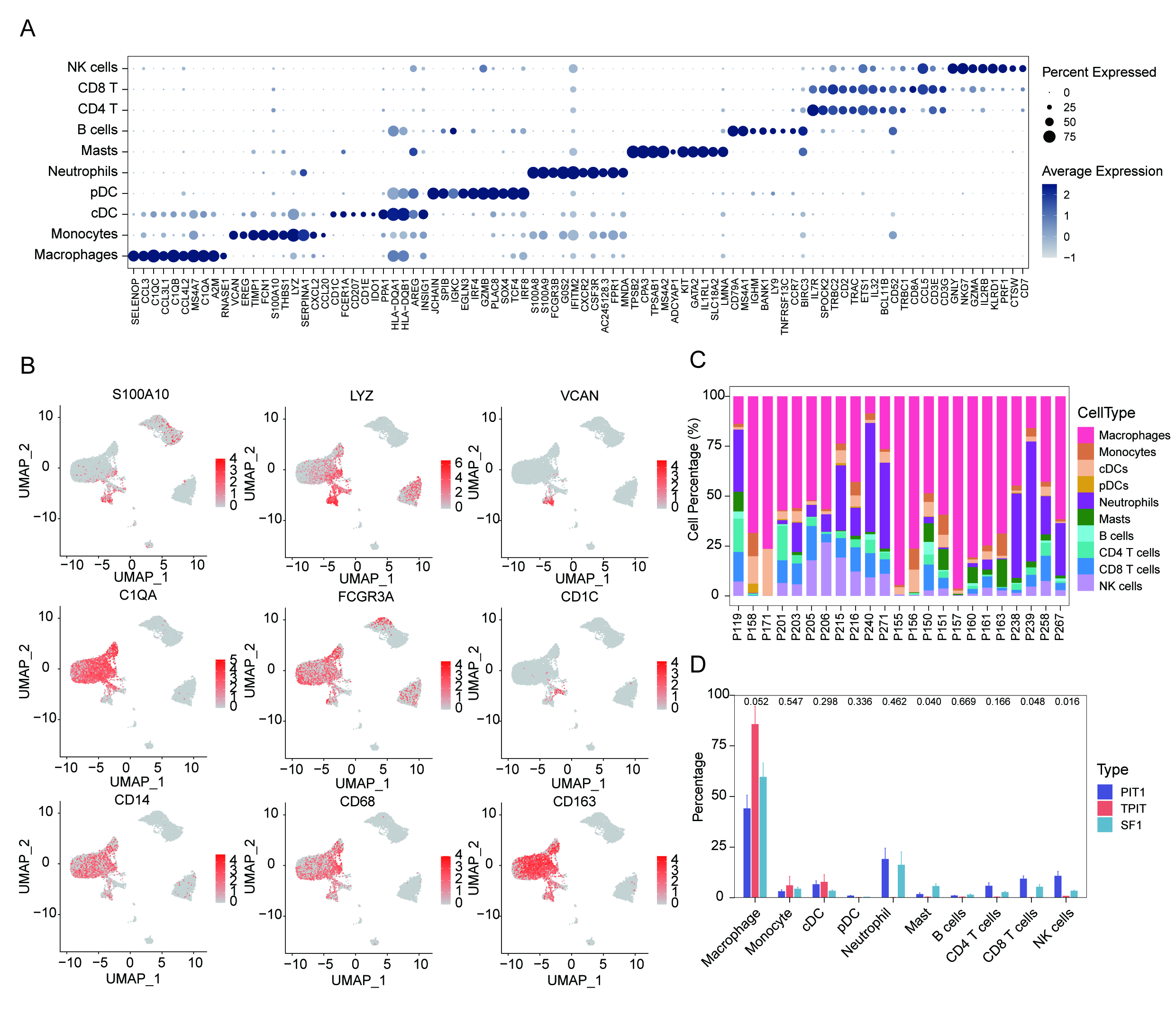


**Figure S4**. **A**. Dot plot depicting the expression level of the specific marker genes for the 10 subtypes of immune cells. **B.** UMAP map showing the expression feature of the marker gene for each immune cell type. **C-D**. The bar plot shows the immune cell percentage for each sample (**C**) and 3 lineages (**D**). P-values were calculated using calculated using ANOVA.


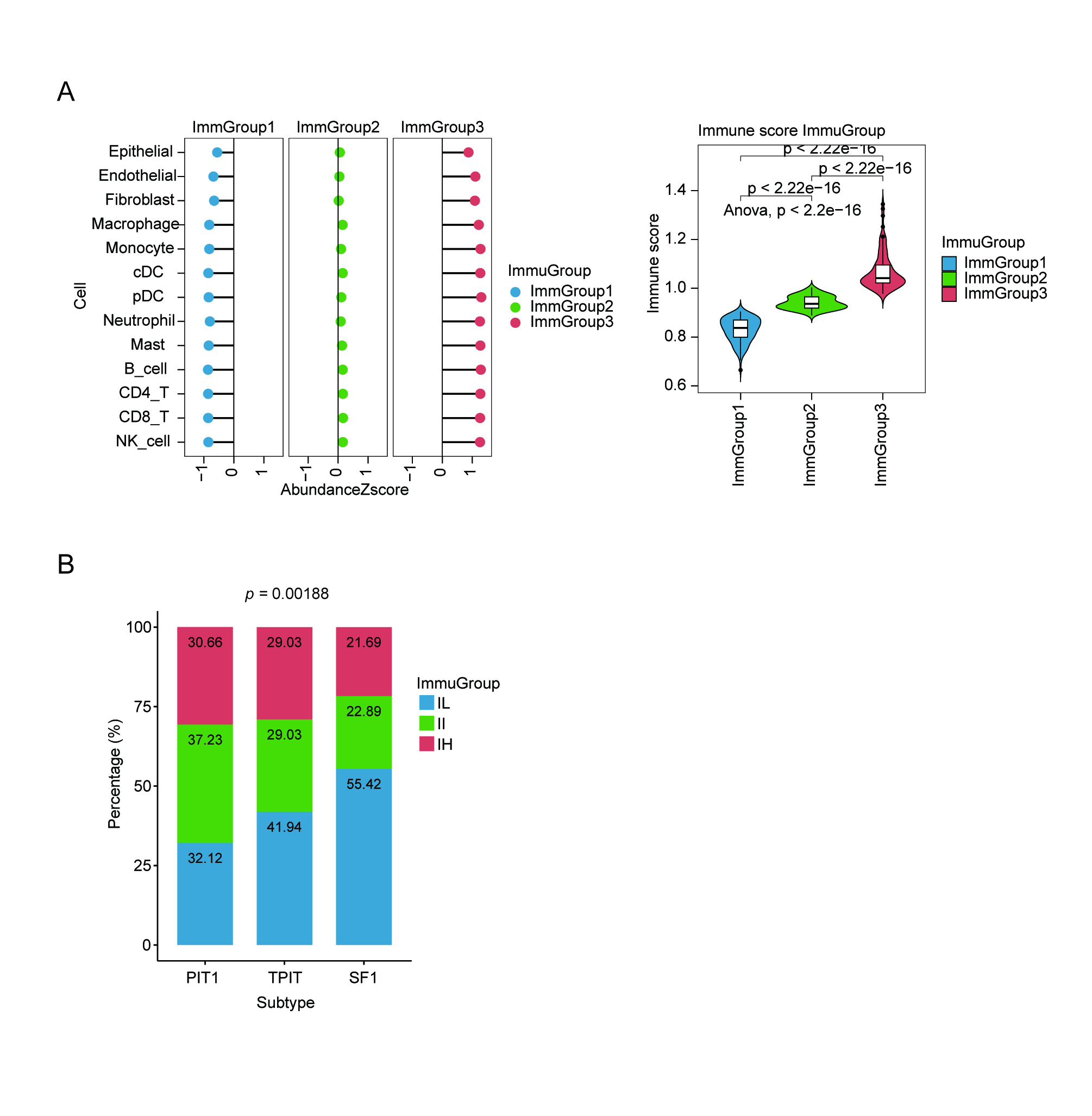


**Figure S5**. **A.** The overview reveals the abundance score of 13 immune cells from three lineages (left) or three immune groups (right). **B.** the composition of immune classification in the three lineages of tumor cells. P-values were calculated using calculated using ANOVA.


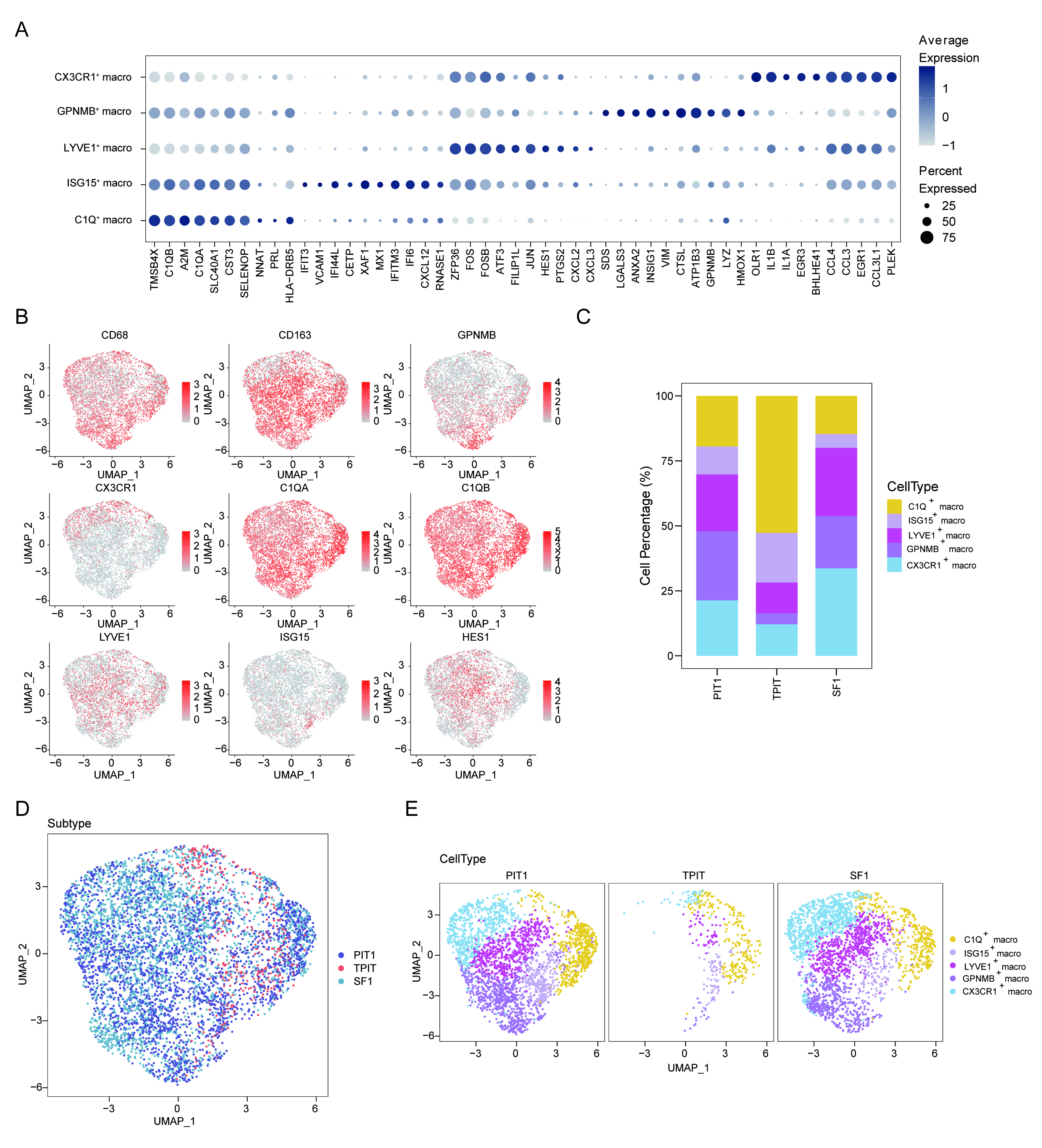
 **Figure S6**. **A.** Dot plot depicting the expression level of the specific marker genes for the 5 subgroups of macrophages. **B.** UMAP map showing the expression feature of the marker gene for each macrophage. **C.** The bar plot shows the cell percentage of 5 subgroups of macrophages for each lineage. **D**. UMAP map of the immune cells color-coded by 3 lineages. **E.** UMAP map of the immune cells for each lineage color-coded by 5 subgroups of macrophages.


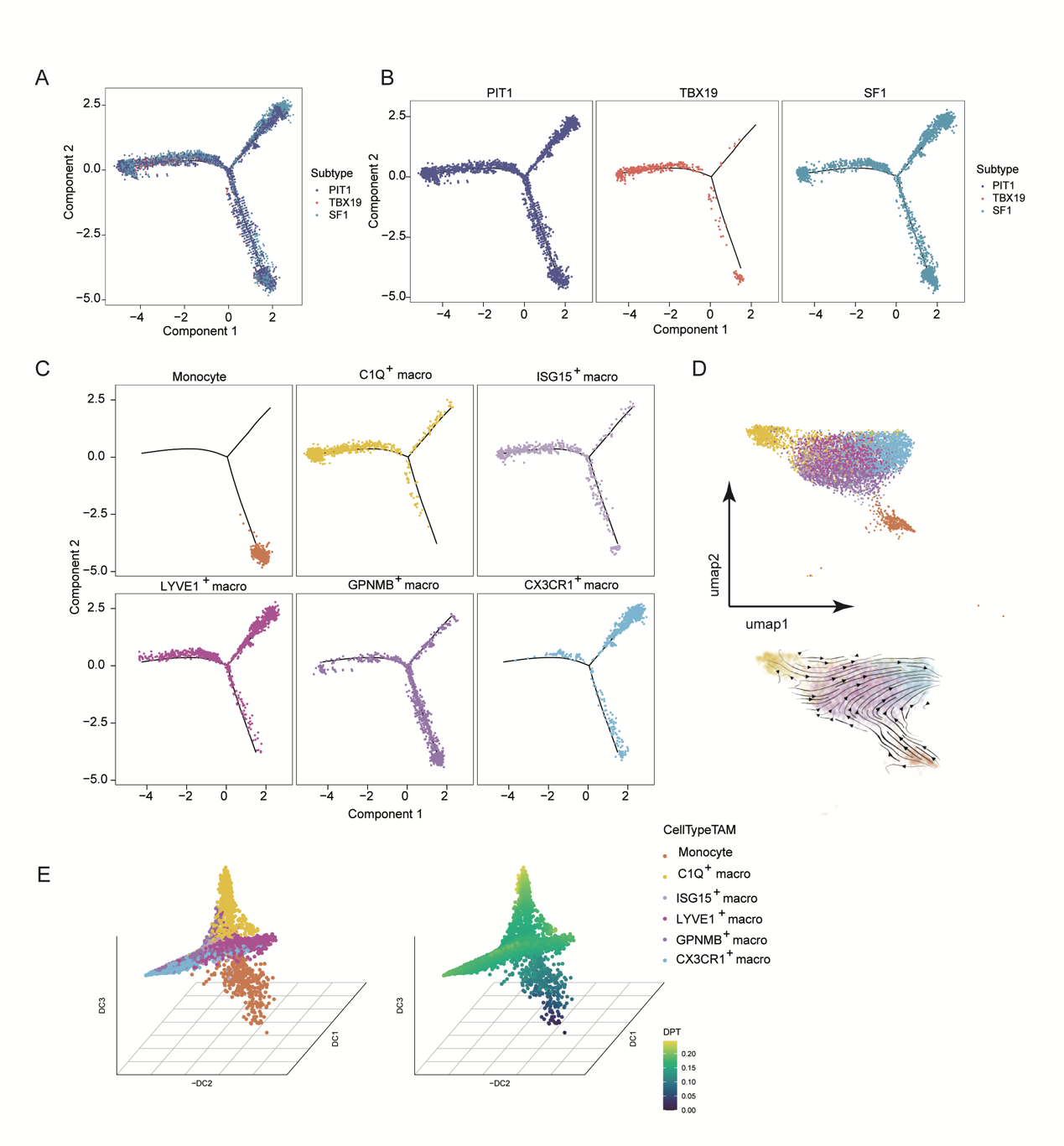


**Figure S7**. **A-C.** The monocle2 trajectory plot of monocytes and the 5 subtypes of macrophages color-coded by three lineages (**A**). The percentage of the cells on each branch for different lineages was shown (**B**). The monocle2 trajectory plot of monocytes and the 5 subtypes of macrophages was shown individually (**C**). **D**. The trajectory plot of monocyte and the 5 subtypes of macrophages predicted by scTour. **E**. The trajectory plot of monocyte and the 5 subtypes of macrophages indicated by Diffusion Map.


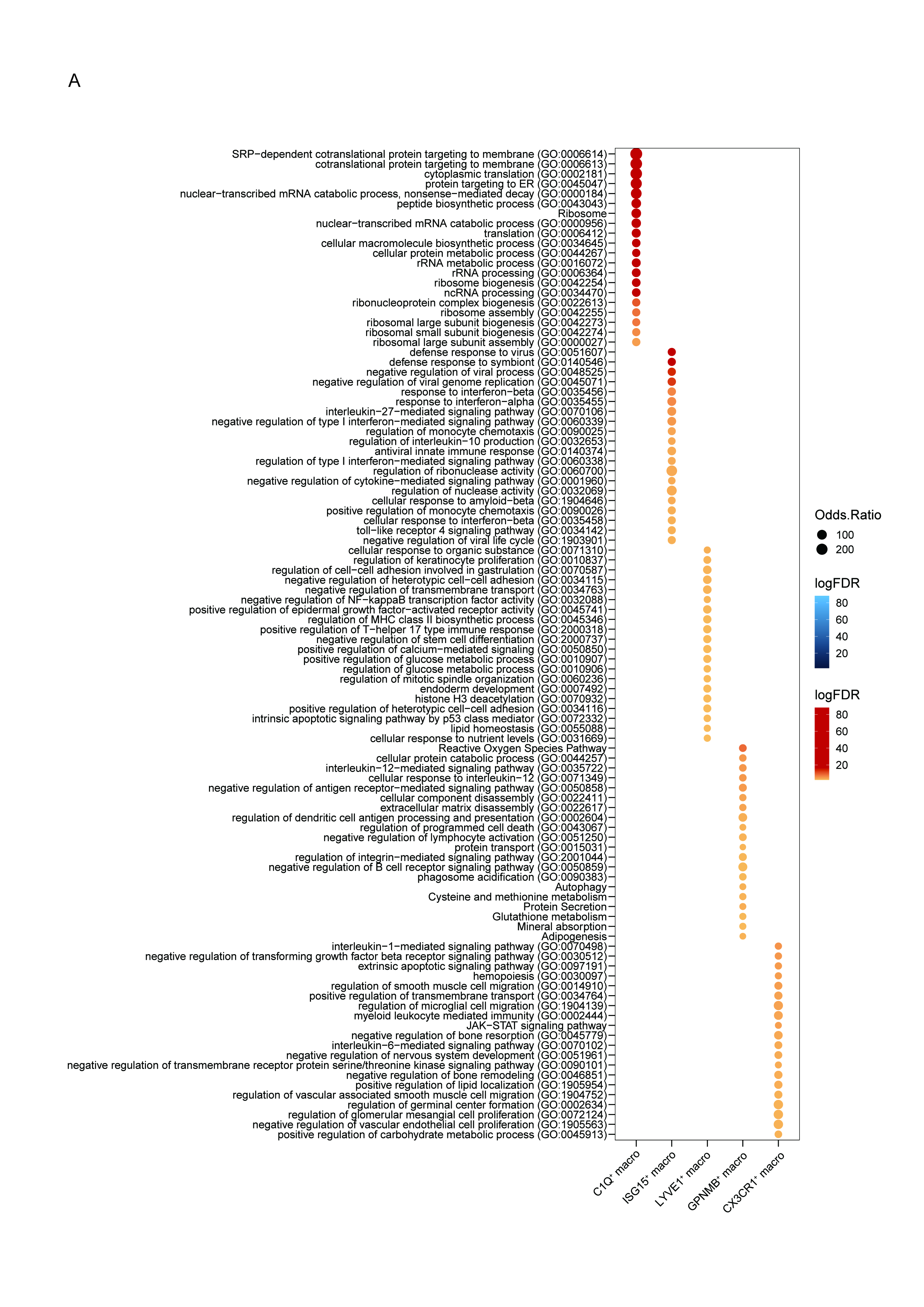


**Figure S8**. A bubble diagram shows the Gene Ontology (GO) biological process (BP) in terms of 5 distinct subgroups of macrophages.


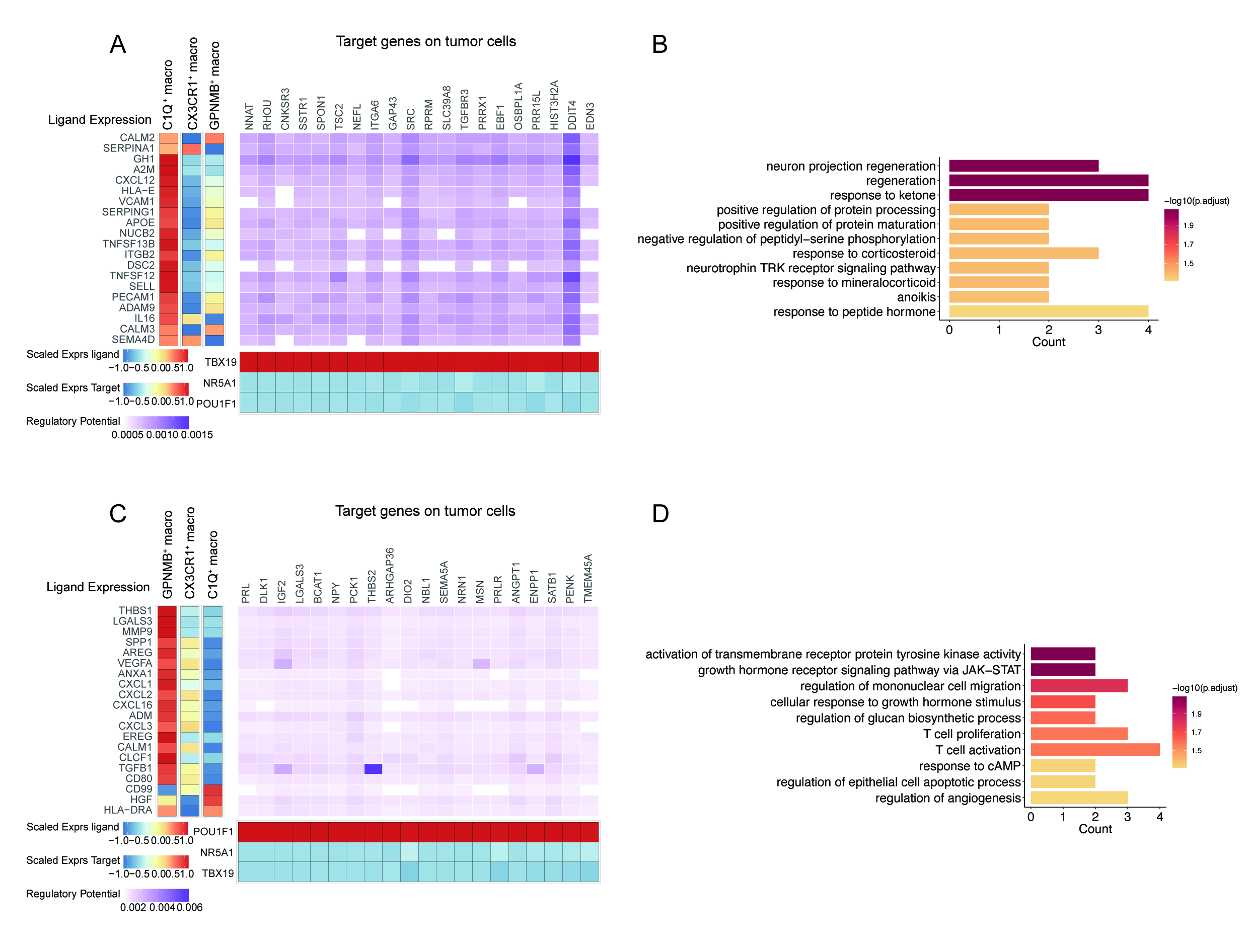


**Figure S9**. **A**. Heatmap showing the ligand expressed highly in the *GPNMB^+^* macrophages (left) and the expression level of the target gene on *POU1F1^+^* tumor cells (right). **B.** The signal pathway is enriched from the ligand-receptor interaction between *GPNMB^+^* macrophages and *POU1F1^+^* tumor cells. **C.** Heatmap showing the ligand expressed high in the *C1Q^+^* macrophages (left) and the expression level of the target gene on *TBX19^+^* tumor cells (right). **D.** The signal pathway enriched from the ligand-receptor interaction between *C1Q^+^* macrophages and *TBX19^+^* tumor cells.


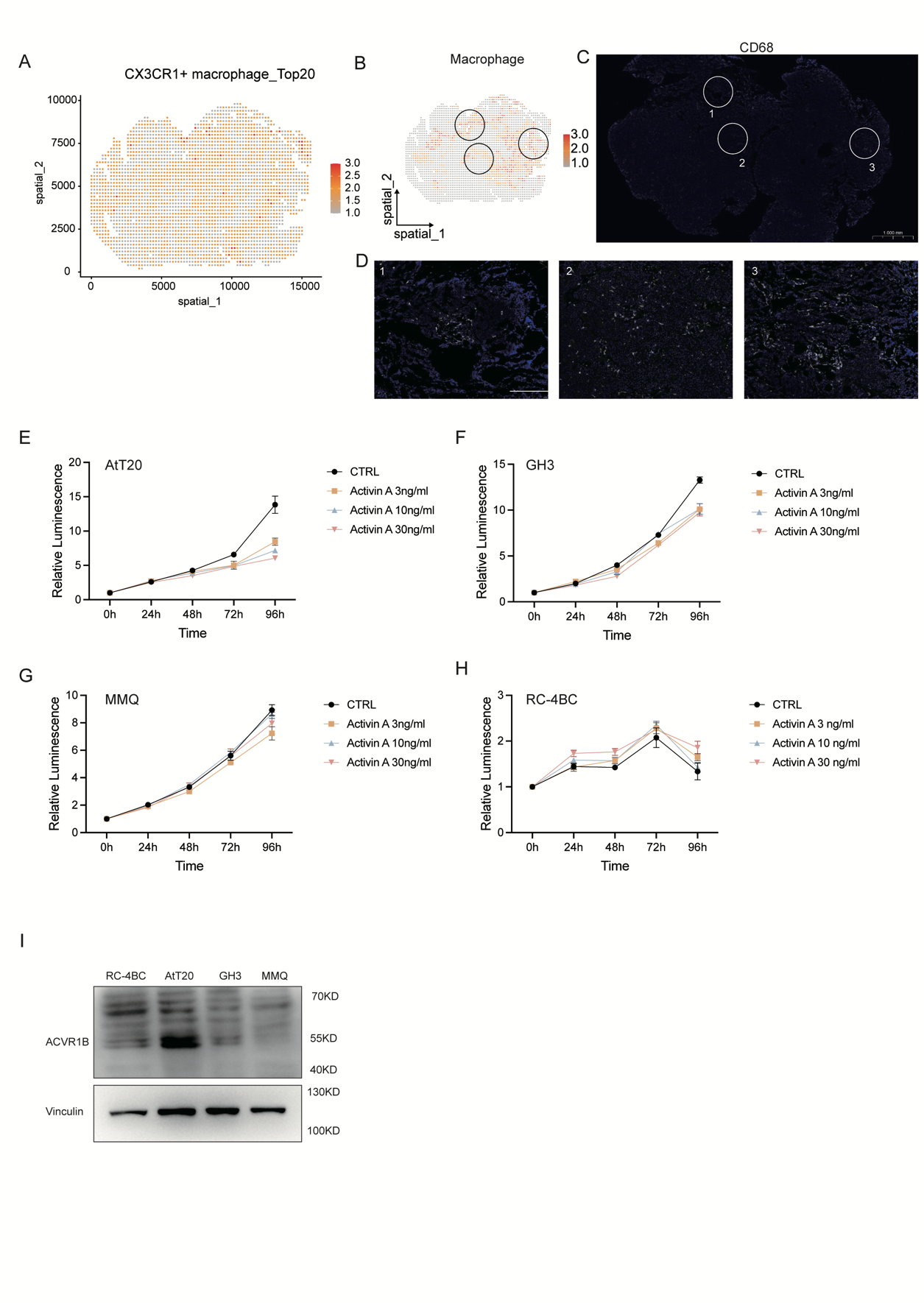


**Figure S10**. **A**. Spatial feature plot shows the module scores of *CX3CR1^+^* macrophages in the ST sample. **B-D.** Spatial feature plot shows the location and proportions of macrophages in ST spots using deconvolution results **(B),** immunofluorescence reveals the macrophage (CD68) localize on a serial section of the spatially sequenced tumour (C-D). The circle represents the enlarged area, presented in Figure S10D. Scared bar as indicated. **E-H.** The cell viability in the AtT20, GH3, MMQ, and RC-4BC cell lines was treated with activin A (10 ng/ml). **I.** The relative protein expression of ACVR1B in the AtT20, GH3, MMQ, and RC-4BC cell lines.


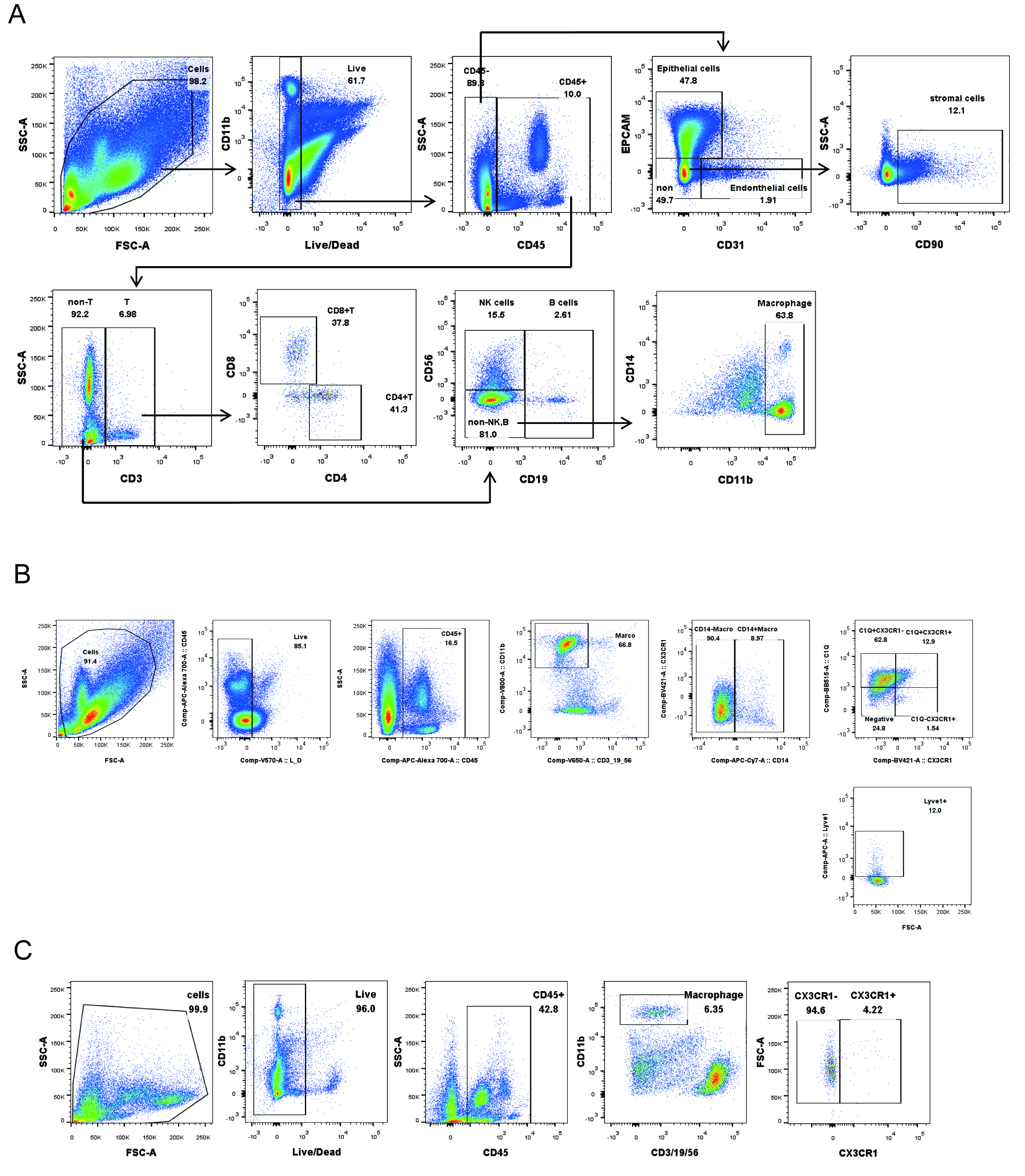


**Figure S11**. Gating strategy of flow cytometry.
