# Supplementary material for: Single-cell transcriptomics reveal distinct immune-infiltrating phenotypes and macrophage–tumor interaction axes among different lineages of pituitary neuroendocrine tumors
Source: Genome Med. 2024 Apr 24;16:60. doi: 10.1186/s13073-024-01325-4 (PMC11040908; doi:10.1186/s13073-024-01325-4)
Supplement: Supplementary file 2 — Additional file 2. Table S1-5. Clinical information of Bulk-RNA seq (Table S1), scRNA-seq (Table S2), TMA (Table S3), FC (Table S4) and clinical information for experiments (Table S5). Table S6. Quality control and filtration. Table S7. Spatial feature expression plots of ACVR1B coexpressing with INHBA and in adjacent spot. Table S8. List of antibodies and chemical dyes. Table S9. qPCR Oligonucleotide Sequences. Table S10. Cell type-specific markers in the UMAP representation of PitNETs. Table S11. Specific markers of macrophages. [file 13073_2024_1325_MOESM2_ESM.zip › Additional file 2/Supplementary table S1.zip!/Supplementary Table s8-antibody.docx]

**Supplementary Table 8**. List of antibodies and chemical dyes used in this article

| Name of antibody | Target | Vendor | Cat. | Dilution | Application |
| --- | --- | --- | --- | --- | --- |
| Fractalkine Receptor Polyclonal Antibody | CX3CR1 | Immunoway | YT5112 | 1:100 | IF |
| Human/Mouse/Rat Activin A beta A subunit Antibody  Anti-CD68 antibody (ab125212) | ACTIVIN A  CD68 | R&D Systems  Abcam | AF338  Ab125212 | 1:100  1:100 |  |
| Fluorescein (FITC)–conjugated Affinipure Donkey Anti-Goat IgG(H+L) | Goat IgG | Proteintech | SA00003-3 | 1:100 |  |
| CoraLite594 – conjugated Donkey Anti-Rabbit IgG(H+L) | Rabbit IgG | Proteintech | SA00013-8 | 1:100 |  |
| Hoechst 33342  Cleaved Caspase-3 (Asp175) (5A1E)  Ki-67 (D3B5) Rabbit mAb | Nuclear  Cleaved-caspase 3  Ki-67 | Yeason  Cell Signaling  Cell Signaling | 40731ES10  9664  9129 | 1:1000  1:2000  1:400 |  |
| ACTR-IB Polyclonal Antibody | ACVR1B | Immunoway | YT0106 | 1:1000 | WB |
| β-Actin (8H10D10) Mouse mAb | β-Actin | Cell Signaling | 3700 | 1:1000 |  |
| Mouse Anti-Rabbit IgG (Light-Chain Specific) (D4W3E) mAb (HRP Conjugate) | Rabbit IgG | Cell Signaling | 93702 | 1:5000 |  |
| Goat Anti-Mouse IgG, Light-Chain Specific Antibody (HRP Conjugate) | Mouse IgG | Cell Signaling | 91196S | 1:5000 |  |
| PE/Cyanine7 anti-human CD68 Antibody | CD68 | Biolegend | 333816 | 1:100 | Flow cytometry and sorting |
| Brilliant Violet 785™ anti-mouse/human CD11b Antibody | CD11b | Biolegend | 101243 | 1:100 |  |
| Alexa Fluor® 700 anti-human CD45 | CD45 | Biolegend | 368514 | 1:100 |  |
| BV650 Mouse Anti-Human CD3(SK7 ) | CD3 | BD Biosciences | 563999 | 1:100 |  |
| BV650 Mouse Anti-Human CD19(SJ25C1 ) | CD19 | BD Biosciences | 563226 | 1:100 |  |
| BV650 Mouse Anti-Human CD56(NCAM16.2 ) | CD56 | BD Biosciences | 564057 | 1:100 |  |
| APC-Cy7 Mouse Anti-Human CD14(MφP9) | CD14 | BD Biosciences | 557831 | 1:100 |  |
| BD Horizon™ BUV737 Mouse Anti-Human CD16 | CD16 | BD Biosciences | 612786 | 1:100 |  |
| BD Pharmingen™ PE-Cy™5 Mouse Anti-Human HLA-DR | HLA-DR | BD Biosciences | 551375 | 1:100 |  |
| PE anti-human Folate Receptor β (FR-β) Antibody | Folate Receptor | Biolegend | 391704 | 1:100 |  |
| APC anti-human Tim-4 Antibody | Tim-4 | Biolegend | 354008 | 1:100 |  |
| LYVE1 Polyclonal Antibody, DyLight™ 488 | LYVE1 | Invitrogen | PA5-22783 | 1:100 |  |
| PE/Cyanine7 anti-human CD88 (C5aR) Antibody | CD88 | Biolegend | 344308 | 1:100 |  |
| BD OptiBuild™ BV510 Mouse Anti-Human CD89 | CD89 | BD Biosciences | 744375 | 1:100 |  |
| PE Mouse Anti-Human CD11c(3.9) | CD11c | BD Biosciences | 565910 | 1:100 |  |
| Brilliant Violet 605™ anti-human CD163 Antibody | CD163 | Biolegend | 333616 | 1:100 |  |
| BV421 Rat Anti-Human CX3CR1(2A9-1) | CX3CR1 | BD Biosciences | 565800 | 1:100 |  |
| PerCP/Cyanine5.5 anti-human CD206 (MMR) Antibody | CD206 | Biolegend | 321122 | 1:100 |  |
| Alexa Fluor® 700 Mouse Anti-Human CD45  PerCP/Cyanine5.5 anti-human CD3 Antibody  BUV496 Mouse Anti-Human CD4  BUV805 Mouse Anti-Human CD8  Brilliant Violet 650™ anti-human CD19 Antibody  PE-CF594 Mouse Anti-Human CD56  BV786 Mouse Anti-Human CD11b  APC-Cy™7 Mouse Anti-Human CD14  PE/Cyanine7 anti-human CD31 Antibody  APC anti-human CD326 (EpCAM) Antibody  Brilliant Violet 510™ anti-human CD90 (Thy1) Antibody  BV421 Mouse Anti-Human CD25  PE-Cy™5 Mouse Anti-Human CD127  Brilliant Violet 605™ anti-human Ki-67 Antibody  FITC-conjugated Annexin V  PI solution | CD45  CD3  CD4  CD8  CD19  CD56  CD11b  CD14  CD31  EpCAM  CD90  CD25  CD127  Ki67  Phosphatidylserine  Nuclear | BD Biosciences  Biolegend  BD Biosciences  BD Biosciences  Biolegend  BD Biosciences  BD Biosciences  BD Biosciences  Biolegend  Biolegend  Biolegend  BD Biosciences  BD Pharmingen™  Biolegend  Dojindo  Dojindo | 560566  300328  612936  749366  363026  564849  742642  557831  303117  324208  328126  562442  624350  350521  AD10  AD10 | 1：100  1：100  1：100  1：100  1：100  1：100  1：100  1：100  1：100  1：100  1：100  1：100  1：100  1：100  1：100  1：50 |  |
| Zombie Yellow™ Fixable Viability Kit | Live and Dead | Biolegend | 423103 | 1：1000 |  |
