# Supplementary material for: Single-cell transcriptomics reveal distinct immune-infiltrating phenotypes and macrophage–tumor interaction axes among different lineages of pituitary neuroendocrine tumors
Source: Genome Med. 2024 Apr 24;16:60. doi: 10.1186/s13073-024-01325-4 (PMC11040908; doi:10.1186/s13073-024-01325-4)
Supplement: Supplementary file 2 — Additional file 2. Table S1-5. Clinical information of Bulk-RNA seq (Table S1), scRNA-seq (Table S2), TMA (Table S3), FC (Table S4) and clinical information for experiments (Table S5). Table S6. Quality control and filtration. Table S7. Spatial feature expression plots of ACVR1B coexpressing with INHBA and in adjacent spot. Table S8. List of antibodies and chemical dyes. Table S9. qPCR Oligonucleotide Sequences. Table S10. Cell type-specific markers in the UMAP representation of PitNETs. Table S11. Specific markers of macrophages. [file 13073_2024_1325_MOESM2_ESM.zip › Additional file 2/Supplementary table S1.zip!/Supplementary Table s9.docx]

**Supplementary Table 9**. qPCR Oligonucleotide Sequences.

| Gene | Forward | Reverse | Source |
| --- | --- | --- | --- |
| BTG2 | ACGGGAAGGGAACCGACAT | CAGTGGTGTTTGTAGTGCTCTG | Primerbank |
| EGR2 | TCAACATTGACATGACTGGAGAG | AGTGAAGGTCTGGTTTCTAGGT |  |
| EGR3 | GACATCGGTCTGACCAACGAG | GGCGAACTTTCCCAAGTAGGT |  |
| NR0B1 | CCGCTTGCAGTTCGAGACT | CTGATGTTCAGACTCCAGCATTT |  |
| SDC4 | GGACCTCCTAGAAGGCCGATA | AGGGCCGATCATGGAGTCTT |  |
| ACTB | CATGTACGTTGCTATCCAGGC | CTCCTTAATGTCACGCACGAT |  |
